# Supplementary material for: Characterization and individual-level prediction of cognitive state in the first year after ‘mild’ stroke
Source: PLoS One. 2024 Aug 30;19(8):e0308103. doi: 10.1371/journal.pone.0308103 (PMC11364298; doi:10.1371/journal.pone.0308103)
Supplement: S1 Table — (DOCX) [file pone.0308103.s001.docx]

| **T****able S1. Baseline characteristics of Australian and Singapore stroke cohorts for common variables across datasets** | | | | | |
| --- | --- | --- | --- | --- | --- |
| **Variables** | **Levels** | **Singapore**  **(n=191)** | **Australia**  **(n=119)** | **Estimate (95% C.I.)** | **p-value*** |
| Sex | Female | 64 (33.51%) | 37 (31.09%) | 1.12 (0.67 to 1.89) | 0.709 |
|  | Male | 127 (66.49%) | 82 (68.91%) |  |  |
| Hypertension | No | 56 (29.32%) | 56 (47.06%) | 0.47 (0.28 to 0.77) | **0.002** |
|  | Yes | 135 (70.68%) | 63 (52.94%) |  |  |
| Ischemic heart disease | No | 166 (86.91%) | 97 (81.51%) | 1.5 (0.76 to 2.95) | 0.254 |
|  | Yes | 25 (13.09%) | 22 (18.49%) |  |  |
| Diabetes | No | 120 (62.83%) | 102 (85.71%) | 0.28 (0.15 to 0.52) | **<.001** |
|  | Yes | 71 (37.17%) | 17 (14.29%) |  |  |
| Previous stroke | No | 158 (82.72%) | 106 (89.08%) | 0.59 (0.27 to 1.21) | 0.141 |
|  | Yes | 33 (17.28%) | 13 (10.92%) |  |  |
| Depression** | No | 173 (90.58%) | 92 (77.31%) | 2.87 (1.41 to 5.94) | **0.002** |
|  | Yes | 17 (8.9%) | 26 (21.85%) |  |  |
| Current smoker | No | 119 (62.3%) | 100 (84.03%) | 0.3 (0.16 to 0.55) | **<.001** |
|  | Yes | 72 (37.7%) | 18 (15.13%) |  |  |
| Ethnicity | Ethnic majority | 129 (67.54%) | 72 (60.5%) | 1.36 (0.82 to 2.25) | 0.223 |
|  | Other | 62 (32.46%) | 47 (39.5%) |  |  |
| Disability | No disability | 171 (89.53%) | 102 (85.71%) | 1.42 (0.67 to 3.01) | 0.368 |
|  | Some disab. | 20 (10.47%) | 17 (14.29%) |  |  |
| Marital status | Married | 146 (76.44%) | 80 (67.23%) | 1.58 (0.92 to 2.71) | 0.088 |
|  | Not married | 45 (23.56%) | 39 (32.77%) |  |  |
| Charlson cmb. index | Median (IQR) | 3 (1) | 3 (2) | 0 (0 to 0) | 0.351 |
| Age (years) | Median (IQR) | 59.59 (12.31) | 67.8 (15.95) | -6.635 (-9.238 to -4.006) | **<.001** |
| NIHSS (total score) | Median (IQR) | 3 (3) | 2 (3) | 1 (0 to 1) | **<.001** |
| Onset to study enrolment (days) | Median (IQR) | 0 (1) | 3.34 (2.31) | -3.07 (-3.28 to -2.84) | **<.001** |
| *p-values from Fisher and Wilcoxon rank-sum tests (as appropriate); ** Depression at admission based on Patient Health Questionnaire (PHQ-2) for Australia; and the Centers for Epidemiological Studies Depression (CES-D > 15 points) scale for Singapore; **Cmb**= Comorbidity; **IQR**=Interquartile range | | | | | |
