# Supplementary material for: Characterization and individual-level prediction of cognitive state in the first year after ‘mild’ stroke
Source: PLoS One. 2024 Aug 30;19(8):e0308103. doi: 10.1371/journal.pone.0308103 (PMC11364298; doi:10.1371/journal.pone.0308103)
Supplement: S2 Table — (DOCX) [file pone.0308103.s002.docx]

| **Table S2. Changes in MoCA scores from baseline to 3- and 12-months post-stroke for group 3 “Improved-declined” (n=45)^*^ in START cohort** | | | | | | | | | |
| --- | --- | --- | --- | --- | --- | --- | --- | --- | --- |
| **Item (range)** | **Baseline Median (IQR)** | **3 Months Median (IQR)** | **z-val.1**  **(95% CI)** | **Change 1**  **(p-value)** | **12 Months Median (IQR)** | **z-val 2**  **(95% CI)** | **Change 2**  **(p-value)** | **z-val 3**  **(95% CI)** | **Overall change**  **(p-value)** |
| **Total score**  **(0-30 points)** | **24 (6)** | **28 (3)** | **-4 (-5, -3)** | **<.001** | **25 (6)** | **2.5 (2, 3.5)** | **<.001** | -1 (-2, 0) | **0.017** |
| **Exec/Visuosp**  **(0-5 points)** | **4 (3)** | **5 (1)** | **-1 (-1.5, -0.5)** | **<.001** | **4 (2)** | **0.5 (0.5, 1)** | **0.001** | 0 (-0.5, 0.5) | 0.715 |
| Naming  (0-3 points) | 3 (0) | 3 (0) | 0 (0, 0) | 0.173 | 3 (0) | 0 (-Inf, 0) | 0.25 | 0 (0, 0) | 0.537 |
| **Attention**  **(0-6 points)** | **10 (2)** | **10 (1)** | **-0.5 (-1, 0)** | **0.047** | 9 (2) | 0.5 (0, 0.5) | 0.146 | 0 (-1, 0) | 0.304 |
| **Language**  **(0-3 points)** | **5 (2)** | **6 (1)** | **-0.5 (-1, -0.5)** | **<.001** | **5 (2)** | **0.5 (0.5, 1)** | **<.001** | 0 (-0.5, 0.5) | 0.874 |
| **Abstraction**  **(0-2 points)** | **2 (1)** | **2 (0)** | **0 (-0.5, 0)** | **0.016** | 2 (1) | 0 (0, 0) | 0.276 | 0 (-0.5, 0) | 0.415 |
| **Delayed recall**  **(0-5 points)** | **2 (1)** | **4 (2)** | **-1.5 (-2, -1)** | **<.001** | **3 (2)** | **0.5 (0.5, 1)** | **<.001** | -1 (-1, -0.48) | **0.003** |
| **Orientation**  **(0-6 points)** | 6 (0) | 6 (0) | 0 (0, 0) | 0.062 | **6 (1)** | **0 (0, 0.5)** | **0.016** | 0 (0, 0.5) | 0.631 |
| ^*^ One patient removed due to severe aphasia at baseline.  All p-values obtained from Asymptotic Wilcoxon-Pratt signed-rank test. **Change 1**=comparison of MoCA at baseline and 3-months post-stroke; **Change 2**=comparison of MoCA at 3- and 12-months post-stroke; **Overall change**=change from baseline to 12-months.  **Exec/visuosp**=executive and visuospatial function; **IQR**=Interquartile range.  **MoCA**=Montreal Cognitive Assessment. | | | | | | | | | |
