# Supplementary material for: Characterization and individual-level prediction of cognitive state in the first year after ‘mild’ stroke
Source: PLoS One. 2024 Aug 30;19(8):e0308103. doi: 10.1371/journal.pone.0308103 (PMC11364298; doi:10.1371/journal.pone.0308103)
Supplement: S3 Table — (DOCX) [file pone.0308103.s003.docx]

| **Table S3. Exploratory, unadjusted regression analyses predicting Montreal Cognitive Assessment (MoCA) using baseline variables in START cohort (n=119)** | | | | |
| --- | --- | --- | --- | --- |
| **Dependent variable (MoCA)** | **Time-point** | **Baseline predictor** | **Estimate (95% CI)^a^** | **p-value^b^** |
|  |  |  | **Bivariate binary logistic regression** | |
| Cognitive impairment  (MoCA < 24) | Baseline (day 3-7) | Secondary education or more | 0.125 (0.033 to 0.478) | **0.002** |
|  | 3 months | Ethnicity (other) | 10 (1.940 to 51.542) | **0.006** |
|  |  | Ever smoking (yes) | 8.662 (1.109 to 67.638) | **0.04** |
|  |  | MoCA score | 0.688 (0.551 to 0.858) | **0.001** |
|  | 12 months | Ethnicity (other) | 8.437 (1.624 to 43.843) | **0.011** |
|  |  | Ever smoking (yes) | -2 (-3.8 to -0.2) | **0.031** |
|  |  | MoCA score | 0.561 (0.397 to 0.795) | **0.001** |
|  |  |  | **Bivariate quantile regression** | |
| MoCA median score | Baseline (day 3-7) | Secondary education or more | 5 (2.709 to 7.291) | **<.001** |
|  |  | Some disability (mRS 1–2 points) | -3 (-5.787 to -0.213) | **0.037** |
|  | 3 months | Secondary education or more | 2 (0.367 to 3.633) | **0.018** |
|  |  | RAPA strength score | 0.667 (0.065 to 1.268) | **0.032** |
|  |  | MoCA score | 0.5 (0.366 to 0.634) | **<.001** |
|  | 12 months | Secondary education or more | 3.176 (0.146 to 6.207) | **0.042** |
|  |  | Age | -0.078 (-0.126 to -0.029) | **0.002** |
|  |  | RAPA strength score | 1 (0.155 to 1.845) | **0.022** |
|  |  | MoCA score | 0.587 (0.382 to 0.791) | **<.001** |
|  |  |  | **Bivariate mixed quantile regression** | |
| MoCA median score | Longitudinal analysis | Secondary education or more | 4 (1.423 to 6.577) | **0.003** |
|  |  | Ischemic heart disease | -2 (-3.946 to -0.054) | **0.044** |
|  |  | Age | -0.087 (-0.134 to -0.04) | **0.001** |
|  |  | RAPA strength score | 0.667 (0.090 to 1.244) | **0.024** |
|  |  |  | **Bivariate mixed Gamma regression** | |
| MoCA Gamma score | Longitudinal analysis | Age | 1.174 (0.3 to 2.048) | **0.008** |
|  |  | NIHSS score | 0.986 (0.132 to 1.839) | **0.024** |
|  |  | RAPA strength score | -0.94 (-1.844 to -0.036) | **0.042** |
| **mRS**=modified Rankin Scale; **RAPA**=Rapid Assessment of Physical Activity; **NIHSS**=National Institutes of Health Stroke Scale; **MoCA**=Montreal Cognitive Assessment  **Note:** Variables tested in all model formulations included sex, dichotomized education (less than secondary / secondary or more), previous stroke, previous TIA, hypertension, atrial fibrillation, diabetes, ischemic heart disease, pre-morbid disability (mRS), ethnicity (Australia/NZ or other), age, stroke severity (NIHSS), depression (MADRS), aerobic and strength capacity (RAPA), and body mass index (kg/m^2^). *Estimates represent slope of linear relationships (quantile regression and quantile mixed regression), and odds ratio, (binary logistic regression). | | | | |
