# Supplementary material for: Characterization and individual-level prediction of cognitive state in the first year after ‘mild’ stroke
Source: PLoS One. 2024 Aug 30;19(8):e0308103. doi: 10.1371/journal.pone.0308103 (PMC11364298; doi:10.1371/journal.pone.0308103)
Supplement: S4 Table — (DOCX) [file pone.0308103.s004.docx]

| **Table S4. Trajectory profiles of stroke clusters at baseline, 3- and 12-months post-stroke in START cohort, based on changes in MoCA scores** | | | | | | | | | | |
| --- | --- | --- | --- | --- | --- | --- | --- | --- | --- | --- |
| **Variable*** | **1.overall improver** | **2.improved-stable** | **3.improved-declined** | **4.stable-improved** | **5.overall stable** | **6.stable-declined** | **7.declined-improved** | **8.declined-stable** | **9.overall decliner** |  |
| **n (%)** | 22 (18.49%) | 15 (12.61%) | 46 (38.66%) | 2 (1.68%) | 2 (1.68%) | 7 (5.88%) | 12 (10.08%) | 6 (5.04%) | 7 (5.88%) |  |
| **Males (%)** | 16 (13.45%) | 8 (6.72%) | 32 (26.89%) | 2 (1.68%) | 1 (0.84%) | 6 (5.04%) | 10 (8.4%) | 3 (2.52%) | 4 (3.36%) |  |
| **High-school or more (%)** | 15 (12.61%) | 14 (11.76%) | 39 (32.77%) | 2 (1.68%) | 2 (1.68%) | 7 (5.88%) | 11 (9.24%) | 5 (4.2%) | 7 (5.88%) |  |
| **Marital status (%)** | 15 (12.61%) | 13 (10.92%) | 31 (26.05%) | 0 (0%) | 1 (0.84%) | 5 (4.2%) | 8 (6.72%) | 3 (2.52%) | 4 (3.36%) |  |
| **Disability pre-stroke (%)** | 3 (2.52%) | 1 (0.84%) | 9 (7.56%) | 0 (0%) | 0 (0%) | 1 (0.84%) | 0 (0%) | 3 (2.52%) | 0 (0%) |  |
| **Age (years)** | 22, 66 (13.22) | 15, 62.8 (20.7) | 46, 68.3 (13.35) | 2, 75.05 (6.05) | 2, 61.3 (18.6) | 7, 70.3 (20.45) | 12, 66.05 (14.5) | 6, 72.1 (13.75) | 7, 69.9 (20.05) |  |
| **CharIson Cmb. Index** | 22, 1.5 (2) | 15, 1 (2) | 46, 3 (2) | 2, 2 (1) | 2, 3 (0) | 7, 3 (1) | 12, 3 (2) | 6, 3 (1.5) | 7, 3 (0) |  |
| **NIHSS score w1** | 22, 141.5 (29.75) | 15, 132 (25) | 46, 135 (26.5) | 2, 151.5 (25.5) | 2, 140 (10) | 7, 160 (17) | 12, 141.5 (31) | 6, 145 (20.5) | 7, 136 (13) |  |
| **NIHSS score 3mth** | 22, 77.5 (20) | 15, 75 (15.5) | 46, 75 (10) | 2, 74 (3) | 2, 80 (0) | 7, 85 (13.5) | 12, 79 (7) | 6, 75.5 (11) | 7, 77 (7.5) |  |
| **NIHSS score 12mth** | 22, 128 (13.25) | 15, 122 (20) | 45, 130 (20) | 2, 157 (16) | 2, 100 (5) | 7, 126 (12.5) | 12, 122 (30.5) | 6, 138 (12.25) | 7, 115 (10) |  |
| **Systolic bp w1** | 22, 75.5 (15) | 15, 75 (14.5) | 45, 76 (10) | 2, 89 (4) | 2, 55 (5) | 7, 65 (10.5) | 12, 74 (18.75) | 6, 76 (11) | 7, 68 (9.5) |  |
| **Diastolic bp w1** | 22, 126.5 (18.5) | 15, 125 (13.5) | 46, 125 (14.5) | 2, 147 (23) | 2, 120 (10) | 7, 122 (17) | 12, 124.5 (12.5) | 6, 137 (26) | 7, 120 (4.5) |  |
| **Systolic bp mo3** | 22, 66.5 (20.25) | 15, 73 (10) | 46, 72.5 (12.75) | 2, 64.5 (4.5) | 2, 65 (5) | 7, 70 (7.5) | 12, 75.5 (12.25) | 6, 85 (22) | 7, 67 (5) |  |
| **Diastolic bp mo3** | 22, 22 (3) | 15, 27 (1.5) | 46, 23.5 (6) | 2, 28.5 (0.5) | 2, 27 (3) | 7, 30 (6) | 12, 26.5 (4.25) | 6, 28 (2.25) | 7, 28 (3) |  |
| **Systolic bp 12mth** | 22, 25 (1.75) | 15, 29 (2) | 46, 28 (3) | 2, 28.5 (0.5) | 2, 27 (3) | 7, 30 (6) | 12, 24 (3.25) | 6, 25.5 (4.5) | 7, 26 (1.5) |  |
| **Diastolic bp 12mth** | 22, 27.5 (2.75) | 15, 29 (2) | 46, 24.5 (6) | 2, 30 (0) | 2, 27 (3) | 7, 26 (9.5) | 12, 26 (2.25) | 6, 25.5 (4.5) | 7, 24 (4) |  |
| **RAPA aerobic score w1** | 17, 27 (2) | 14, 28.5 (2.75) | 28, 27.5 (2.25) | 2, 27 (1) |  | 6, 27 (3.75) | 7, 27 (4) | 3, 28 (2.5) | 4, 27 (3.5) |  |
| **RAPA strength score w1** | 17, 28 (1) | 14, 29 (1.75) | 26, 27 (3) | 2, 29 (1) |  | 6, 27.5 (4.75) | 7, 25 (4.5) | 3, 29 (1) | 4, 25 (3.25) |  |
| **RAPA aerobic score 3mth** | 17, 2.4 (0.8) | 14, 2.25 (1.1) | 28, 2.25 (0.92) | 2, 2.65 (0.15) |  | 6, 2.05 (0.65) | 7, 2 (0.45) | 3, 2.2 (0.75) | 4, 1.8 (0.38) |  |
| **RAPA strength score 3mth** | 17, 2.3 (1.2) | 14, 1.95 (0.52) | 26, 2.4 (1.1) | 2, 2.05 (0.05) |  | 6, 1.9 (0.9) | 7, 2.3 (1.03) | 3, 2.1 (0.55) | 4, 1.8 (0.38) |  |
| **RAPA aerobic score 12mth** | 17, 29 (8) | 14, 33 (5.25) | 28, 29 (6) | 2, 28 (7) |  | 6, 31 (9.75) | 7, 27 (4) | 3, 29 (4) | 4, 28 (7.75) |  |
| **RAPA strength score 12mth** | 17, 29 (9) | 14, 32.5 (6) | 26, 27.5 (8.75) | 2, 31.5 (3.5) |  | 6, 31 (11.5) | 7, 29 (6) | 3, 32 (3) | 4, 27 (7.5) |  |
| **MADRS score w1** | 17, 153 (146) | 14, 89 (65.5) | 28, 103 (145.25) | 2, 127.5 (65.5) |  | 6, 85.5 (180) | 7, 77 (65) | 3, 92 (29) | 4, 97 (103.75) |  |
| **MADRS score 3mth** | 17, 120 (163) | 14, 91 (51.5) | 26, 89 (97.5) | 2, 87.5 (40.5) |  | 6, 85 (172.5) | 7, 98 (38) | 3, 62 (31) | 4, 106.5 (97.75) |  |
| **MADRS score 12mth** | 17, 9 (2) | 14, 9.5 (1.75) | 28, 8.5 (2.5) | 2, 9 (0) |  | 6, 11 (1.5) | 7, 10 (3) | 3, 8 (4) | 4, 9 (0.5) |  |
| **MoCA score w1** | 17, 9 (4) | 14, 10 (2) | 26, 8 (1.75) | 2, 10 (0) |  | 6, 11 (1.5) | 7, 10 (3) | 3, 10 (4) | 4, 8.5 (2) |  |
| **MoCA score 3mth** | 17, 7 (1) | 14, 8 (2.75) | 28, 7 (2.25) | 2, 6.5 (0.5) |  | 6, 8 (5.5) | 7, 6 (2.5) | 3, 6 (4) | 3, 7 (3) |  |
| **MoCA score 12mth** | 17, 7 (1) | 14, 8 (1.75) | 26, 7 (2) | 2, 6.5 (0.5) |  | 6, 7.5 (4.75) | 7, 7 (0) | 3, 7 (3.5) | 4, 8 (3) |  |
| **MMSE score 3mth** | 22, 5 (7) | 15, 6 (6) | 44, 2.5 (5.5) | 2, 4 (1) | 2, 11 (5) | 7, 1 (2.5) | 11, 8 (10) | 6, 7 (7.5) | 7, 3 (13.5) |  |
| **MMSE score 12mth** | 22, 3.5 (6.5) | 15, 4 (9.5) | 46, 3.5 (10.5) | 2, 0.5 (0.5) | 2, 12 (0) | 7, 4 (5.5) | 12, 6.5 (9.5) | 6, 4 (0.75) | 7, 4 (5) |  |
| **Stroop ratio 3mth** | 22, 3 (5) | 15, 2 (6) | 43, 6 (12.5) | 2, 0.5 (0.5) | 2, 12 (3) | 7, 5 (5.5) | 12, 4.5 (7) | 6, 2.5 (2.5) | 7, 6 (5) |  |
| **Stroop ratio 12mth** | 22, 2 (2) | 15, 1 (1.5) | 46, 2 (3) | 2, 0.5 (0.5) | 2, 4 (2) | 7, 0 (3.5) | 12, 1 (3) | 6, 0.5 (1) | 7, 1 (3) |  |
| **Ravens score 3mth** | 22, 1 (1) | 15, 0 (1) | 46, 0 (1) | 2, 0 (0) | 2, 0 (0) | 7, 0 (0.5) | 12, 0 (1.25) | 6, 1 (0.75) | 7, 1 (1) |  |
| **Ravens score 12mth** | 22, 0 (1) | 15, 0 (0.5) | 46, 0 (1) | 2, 0 (0) | 2, 0.5 (0.5) | 7, 0 (1) | 12, 0 (1) | 6, 0.5 (1) | 7, 0 (1.5) |  |
| **TMT-B time taken 3mth** | 22, 100 (0) | 15, 100 (0) | 46, 100 (5) | 2, 100 (0) | 2, 100 (0) | 7, 100 (0) | 12, 100 (0) | 6, 100 (0) | 7, 100 (2.5) |  |
| **TMT-B time taken 12mth** | 22, 100 (0) | 15, 100 (0) | 46, 100 (3.75) | 2, 100 (0) | 2, 100 (0) | 7, 100 (0) | 12, 100 (0) | 6, 100 (3.75) | 7, 100 (0) |  |
| **Digit span forward 3mth** | 22, 1 (0.75) | 15, 1 (2) | 46, 1 (1) | 2, 0.5 (0.5) | 2, 1 (0) | 7, 1 (1) | 12, 1 (1) | 6, 1 (0.75) | 7, 1 (0.5) |  |
| **Digit span forward 12mth** | 22, 1 (1) | 15, 0 (1) | 46, 1 (2) | 2, 0 (0) | 2, 1 (0) | 7, 1 (1) | 12, 1 (1) | 6, 1.5 (1.75) | 7, 1 (1) |  |
| **Digit span backward 3mth** | 22, 4 (3) | 15, 3 (1) | 46, 4 (3) | 2, 5 (2) | 2, 5.5 (1.5) | 7, 2 (2.5) | 12, 5.5 (2) | 6, 3.5 (1) | 7, 4 (2) |  |
| **Digit span backward 12mth** | 22, 0 (0.75) | 15, 0 (1.5) | 46, 0 (0.75) | 2, 1.5 (1.5) | 2, 3 (0) | 7, 0 (0) | 12, 0.5 (2) | 6, 0 (0) | 7, 0 (1) |  |
| **Barthel score 3mth** | 22, 5.5 (3.5) | 15, 4 (2) | 45, 4 (3) | 2, 6 (0) | 2, 4.5 (1.5) | 7, 4 (1.5) | 12, 6 (2) | 6, 4 (2.25) | 7, 4 (3) |  |
| **Barthel score 12mth** | 22, 1 (2) | 15, 0 (1) | 45, 0 (1) | 2, 3 (0) | 2, 1.5 (1.5) | 7, 0 (1) | 12, 1 (2) | 6, 1 (2) | 7, 1 (3) |  |
| **mRS score 3mth** | 22, 4 (3.75) | 15, 4 (3) | 46, 3.5 (4) | 2, 6 (1) | 2, 3.5 (0.5) | 7, 3 (2.5) | 12, 4 (3) | 6, 3 (1.5) | 7, 3 (0.5) |  |
| **mRS score 12mth** | 22, 0 (1.75) | 15, 1 (2) | 46, 0 (1) | 2, 1.5 (1.5) | 2, 0.5 (0.5) | 7, 0 (0) | 12, 0 (3) | 6, 0 (0) | 7, 0 (0.5) |  |
| **ACS (RALN) 3mth** | 17, 98 (6) | 14, 96.5 (7.75) | 28, 97 (25.5) | 2, 100 (0) |  | 6, 97 (8) | 7, 89 (22.5) | 3, 94 (11.5) | 4, 93 (12.5) |  |
| **ACS (RALN) 12mth** | 17, 98 (4) | 14, 100 (3.75) | 26, 98 (8.5) | 2, 100 (0) |  | 6, 96 (20.25) | 7, 100 (4) | 3, 100 (8) | 4, 97.5 (9.75) |  |
| **WSAS score 3mth** | 22, 3.5 (8.62) | 15, 8 (10.25) | 45, 2 (11) | 2, 0 (0) | 2, 14.5 (13.5) | 7, 0.5 (8.5) | 12, 2 (5.5) | 6, 4 (4.75) | 7, 12 (15) |  |
| **WSAS score 12mth** | 22, 2 (5.5) | 15, 0 (5.5) | 46, 3.5 (12.5) | 2, 0 (0) | 2, 12.5 (6.5) | 7, 0 (5) | 12, 0 (8.25) | 6, 3.75 (3.38) | 7, 0 (8.5) |  |
| **SIS total 3mo** | 22, 751.87 (67.23) | 14, 746.87 (89.56) | 45, 728.42 (128.64) | 2, 794.44 (2.78) | 2, 688.2 (9.44) | 7, 766.67 (67.86) | 12, 745.24 (66.06) | 6, 719.36 (32.78) | 7, 755.44 (48.53) |  |
| **SIS total 12mo** | 22, 772.22 (60.04) | 15, 766.67 (48.18) | 45, 736.31 (142.56) | 2, 794.44 (5.56) | 2, 631.31 (89.16) | 7, 750.64 (57.68) | 12, 756.85 (70.4) | 6, 681.25 (60.71) | 7, 746.03 (33.17) |  |
| *All continuous variables are reported as **count, median (IQR)**  **ACS**=Activity Card Sort – Retained Activity Level; **BP**=blood pressure; **MADRS**=Montgomery-Åsberg Depression Rating Scale; **MMSE**=Mini-Mental State Examination; **MoCA**=Montreal Cognitive Assessment; **mRS**=Modified Rankin Scale; **NIHSS**=National Institutes of Health Stroke Scale; **RAPA**=Rapid Assessment of Physical Activity; **SIS**=Stroke Impact Scale; **TMT**=Trail Making Test; **w1**= baseline; **WSAS**=Work and Social Adjustment Scale. | | | | | | | | | | |
