# Supplementary material for: Characterization and individual-level prediction of cognitive state in the first year after ‘mild’ stroke
Source: PLoS One. 2024 Aug 30;19(8):e0308103. doi: 10.1371/journal.pone.0308103 (PMC11364298; doi:10.1371/journal.pone.0308103)
Supplement: S5 Table — (DOCX) [file pone.0308103.s005.docx]

| **Table S5. Post-hoc descriptive profile comparison of the two most common cognitive trajectory clusters in START cohort based on MoCA scores** | | | | | | | | | |  |
| --- | --- | --- | --- | --- | --- | --- | --- | --- | --- | --- |
| **Variables** | **1.overall improver** | **3.improved-declined** | **z-value 1** | **95% CI 1** | **p-value 1** | **9.overall decliner** | **z-value 2** | **95% CI 2** | **p-value 2** | |
| **n (%)** | 22 (18.49%) | 46 (38.66%) |  |  |  | 7 (5.88%) |  |  |  | |
| **Males (%)** | 16 (13.45%) | 32 (26.89%) | 0.859 | 0.226, 2.969 | 1 | 4 (3.36%) | 0.513 | 0.063, 4.56 | 0.642 | |
| **High-school or more (%)** | 15 (12.61%) | 39 (32.77%) | 2.559 | 0.584, 11.317 | 0.175 | 7 (5.88%) | Inf | 0.406, Inf | 0.288 | |
| **Marital status, married (%)** | 15 (12.61%) | 31 (26.05%) | 1.036 | 0.312, 3.672 | 1 | 4 (3.36%) | 1.58 | 0.181, 12.466 | 0.665 | |
| **Pre-stroke disability (%)** | 3 (2.52%) | 9 (7.56%) | 1.531 | 0.329, 9.815 | 0.738 | 0 (0%) | 0 | 0, 7.977 | 0.557 | |
| **Age** | 22, 66 (13.22) | 46, 68.3 (13.35) | -0.714 | -8.3, 3.7 | 0.48 | 7, 69.9 (20.05) | -0.051 | -10.4, 17.8 | 0.97 | |
| **Charlson cmb. index** | 22, 1.5 (2) | 46, 3 (2) | -1.269 | -2, 0 | 0.207 | 7, 3 (0) | -1.928 | -2, 0 | 0.058 | |
| **NIHSS score w1** | 22, 2 (2) | 46, 2 (3) | 0.743 | -1, 1 | 0.462 | 7, 1 (3) | 0.233 | -2, 2 | 0.821 | |
| **NIHSS score 3mth** | 22, 1 (1) | 46, 0 (1) | 1.757 | 0, 1 | 0.078 | 7, 1 (1) | -0.383 | -1, 1 | 0.763 | |
| **NIHSS score 12mth** | 22, 0 (1) | 46, 0 (1) | -0.095 | 0, 0 | 0.921 | 7, 0 (1.5) | -0.543 | -1, 0 | 0.59 | |
| **Systolic bp w1** | 22, 141.5 (29.75) | 46, 135 (26.5) | 1.266 | -5, 17 | 0.208 | 7, 136 (13) | 0.204 | -16, 20 | 0.852 | |
| **Diastolic bp w1** | 22, 77.5 (20) | 46, 75 (10) | 1.642 | 0, 12 | 0.102 | 7, 77 (7.5) | 0.818 | -5, 18 | 0.43 | |
| **Systolic bp mo3** | 22, 128 (13.25) | 45, 130 (20) | 0.482 | -5, 10 | 0.635 | 7, 115 (10) | 2.194 | 2, 20 | **0.027** | |
| **Diastolic bp mo3** | 22, 75.5 (15) | 45, 76 (10) | 0.925 | -4, 9 | 0.36 | 7, 68 (9.5) | 1.839 | 0, 19 | 0.067 | |
| **Systolic bp 12mth** | 22, 126.5 (18.5) | 46, 125 (14.5) | 0.493 | -5, 8 | 0.627 | 7, 120 (4.5) | 0.921 | -5, 15 | 0.373 | |
| **Diastolic bp 12mth** | 22, 66.5 (20.25) | 46, 72.5 (12.75) | -1.558 | -11, 1 | 0.121 | 7, 67 (5) | -0.077 | -9, 12 | 0.95 | |
| **RAPA aerobic score w1** | 22, 4 (3) | 46, 4 (3) | -0.027 | -1, 1 | 0.981 | 7, 4 (2) | 0.209 | -2, 2 | 0.851 | |
| **RAPA strength score w1** | 22, 0 (0.75) | 46, 0 (0.75) | 0.254 | 0, 0 | 0.809 | 7, 0 (1) | -0.13 | 0, 0 | 0.883 | |
| **RAPA aerobic score 3mth** | 22, 5.5 (3.5) | 45, 4 (3) | 1.571 | 0, 2 | 0.117 | 7, 4 (3) | 0.655 | -1, 2 | 0.544 | |
| **RAPA strength score 3mth** | 22, 1 (2) | 45, 0 (1) | 1.151 | 0, 1 | 0.261 | 7, 1 (3) | -0.568 | -2, 1 | 0.583 | |
| **RAPA aerobic score 12mth** | 22, 4 (3.75) | 46, 3.5 (4) | 0.742 | -1, 1 | 0.463 | 7, 3 (0.5) | 0.674 | -1, 3 | 0.519 | |
| **RAPA strength score 12mth** | 22, 0 (1.75) | 46, 0 (1) | -0.44 | 0, 0 | 0.666 | 7, 0 (0.5) | 0.422 | 0, 1 | 0.714 | |
| **MADRS score w1** | 22, 5 (7) | 44, 2.5 (5.5) | 0.92 | -1, 4 | 0.362 | 7, 3 (13.5) | -0.437 | -10, 4 | 0.681 | |
| **MADRS score 3mth** | 22, 3.5 (6.5) | 46, 3.5 (10.5) | -0.438 | -3, 2 | 0.666 | 7, 4 (5) | -0.592 | -4, 2 | 0.571 | |
| **MADRS score 12mth** | 22, 3 (5) | 43, 6 (12.5) | -1.845 | -7, 0 | 0.065 | 7, 6 (5) | -1.507 | -6, 1 | 0.14 | |
| **MoCA score w1** | 22, 22 (3) | 46, 23.5 (6) | -1.513 | -4, 0 | 0.132 | 7, 28 (3) | -3.591 | -8, -3 | **0.001** | |
| **MoCA score 3mth** | 22, 25 (1.75) | 46, 28 (3) | -2.885 | -3, -1 | **0.003** | 7, 26 (1.5) | -0.572 | -2, 1 | 0.584 | |
| **MoCA score 12mth** | 22, 27.5 (2.75) | 46, 24.5 (6) | 3.28 | 1, 5 | **0.001** | 7, 24 (4) | 2.547 | 1, 7 | **0.009** | |
| **MMSE score 3mth** | 17, 27 (2) | 28, 27.5 (2.25) | -0.57 | -2, 1 | 0.577 | 4, 27 (3.5) | 0.597 | -1, 6 | 0.609 | |
| **MMSE score 12mth** | 17, 28 (1) | 26, 27 (3) | 0.731 | -1, 2 | 0.474 | 4, 25 (3.25) | 1.318 | -1, 5 | 0.207 | |
| **Stroop ratio 3mth** | 17, 2.4 (0.8) | 28, 2.25 (0.92) | 0.517 | -0.3, 0.6 | 0.613 | 4, 1.8 (0.38) | 1.973 | 0, 1.6 | **0.048** | |
| **Stroop ratio 12mth** | 17, 2.3 (1.2) | 26, 2.4 (1.1) | 0.934 | -0.3, 0.6 | 0.358 | 4, 1.8 (0.38) | 1.709 | -0.1, 1.5 | 0.095 | |
| **Ravens score 3mth** | 17, 29 (8) | 28, 29 (6) | -0.164 | -3, 3 | 0.875 | 4, 28 (7.75) | 0 | -7, 8 | 1 | |
| **Ravens score 12mth** | 17, 29 (9) | 26, 27.5 (8.75) | -0.1 | -3, 3 | 0.926 | 4, 27 (7.5) | -0.18 | -7, 7 | 0.88 | |
| **TMT-B time taken 3mth** | 17, 153 (146) | 28, 103 (145.25) | 1.78 | -1, 85 | 0.076 | 4, 97 (103.75) | 1.03 | -108, 167 | 0.324 | |
| **TMT-B time taken 12mth** | 17, 120 (163) | 26, 89 (97.5) | 0.857 | -23, 62 | 0.399 | 4, 106.5 (97.75) | 0.314 | -97, 146 | 0.782 | |
| **Digit span forward 3mth** | 17, 9 (2) | 28, 8.5 (2.5) | 0.486 | -1, 2 | 0.634 | 4, 9 (0.5) | 0.275 | -1, 2 | 0.789 | |
| **Digit span forward 12mth** | 17, 9 (4) | 26, 8 (1.75) | 0.825 | -1, 2 | 0.42 | 4, 8.5 (2) | 0.412 | -2, 3 | 0.713 | |
| **Digit span backward 3mth** | 17, 7 (1) | 28, 7 (2.25) | 0.764 | -1, 1 | 0.452 | 3, 7 (3) | -0.275 | -5, 2 | 0.869 | |
| **Digit span backward 12mth** | 17, 7 (1) | 26, 7 (2) | 0.013 | -1, 1 | 0.995 | 4, 8 (3) | -0.412 | -3, 2 | 0.715 | |
| **Barthel score 3mth** | 22, 100 (0) | 46, 100 (5) | 0.865 | 0, 0 | 0.405 | 7, 100 (2.5) | 0.433 | 0, 0 | 0.871 | |
| **Barthel score 12mth** | 22, 100 (0) | 46, 100 (3.75) | 0.706 | 0, 0 | 0.491 | 7, 100 (0) | -0.349 | 0, 0 | 0.741 | |
| **mRS score 3mth** | 22, 1 (0.75) | 46, 1 (1) | 0.662 | 0, 1 | 0.513 | 7, 1 (0.5) | -0.307 | -1, 1 | 0.841 | |
| **mRS score 12mth** | 22, 1 (1) | 46, 1 (2) | -1.656 | -1, 0 | 0.101 | 7, 1 (1) | 0.113 | -1, 1 | 1 | |
| **ACS (RALN) 3mth** | 17, 98 (6) | 28, 97 (25.5) | 1.988 | 0, 20 | **0.047** | 4, 93 (12.5) | 1.084 | -4, 20 | 0.303 | |
| **ACS (RALN) 12mth** | 17, 98 (4) | 26, 98 (8.5) | -0.352 | -3, 3 | 0.733 | 4, 97.5 (9.75) | -0.272 | -7, 15 | 0.825 | |
| **WSAS score 3mth** | 22, 3.5 (8.62) | 45, 2 (11) | 0.048 | -3, 2 | 0.965 | 7, 12 (15) | -0.521 | -12, 4 | 0.62 | |
| **WSAS score 12mth** | 22, 2 (5.5) | 46, 3.5 (12.5) | -1.61 | -4.5, 0 | 0.109 | 7, 0 (8.5) | -0.163 | -7, 2 | 0.884 | |
| **SIS total 3mo** | 22, 751.87 (67.23) | 45, 728.42 (128.64) | 0.848 | -20.635, 51.875 | 0.401 | 7, 755.44 (48.53) | -0.051 | -45.764, 44.563 | 0.972 | |
| **SIS total 12mo** | 22, 772.22 (60.04) | 45, 736.31 (142.56) | 1.549 | -4.067, 66.855 | 0.123 | 7, 746.03 (33.17) | 0.892 | -19.305, 49.891 | 0.386 | |
| *All continuous variables are reported as **count, median (IQR).**  **ACS (RALN)**=Activity Card Sort – Retained Activity Level (including new activities); **MADRS**=Montgomery-Åsberg Depression Rating Scale; **MMSE**=Mini-Mental State Examination; **MoCA**=Montreal Cognitive Assessment; **NIHSS**=National Institutes of Health Stroke Scale; **RAPA**=Rapid Assessment of Physical Activity; **SIS**=Stroke Impact Scale; **TMT-B**=Trail Making Test-Part B; **w1**= baseline; **WSAS**=Work and Social Adjustment Scale; z-value 1 and p-value 1 indicate statistical differences between cluster 1 (overall-improvers) and cluster 3 (improved-declined); z-value 2 and p-value 2 indicate statistical differences between cluster 1 (overall-improvers) and cluster 9 (overall decliners); | | | | | | | | | |  |
