# Supplementary material for: Characterization and individual-level prediction of cognitive state in the first year after ‘mild’ stroke
Source: PLoS One. 2024 Aug 30;19(8):e0308103. doi: 10.1371/journal.pone.0308103 (PMC11364298; doi:10.1371/journal.pone.0308103)
Supplement: S6 Table — (DOCX) [file pone.0308103.s006.docx]

| **Table S6. Comparison of START participants on antiplatelet, tPA, or anticoagulant medication (n=31) versus those not on those medications (n=88)** | | | | | | |
| --- | --- | --- | --- | --- | --- | --- |
| **Variables** | **Categories & sample size All \| No med \| On meds** | **All** | **No tPA, anticoagulant or antiplatelet medication (n=88)** | **On tPA, anticoagulant or antiplatelet medication (n=31)** | **Estimate (95% C.I.)** | **p-value** |
| Sex | Female | 37 (42.05%) | 26 (29.55%) | 11 (35.48%) | 0.76 (0.3-2.03) | 0.652 |
|  | Male | 82 (93.18%) | 62 (70.45%) | 20 (64.52%) |  |  |
| Ethnicity | Australian/NZ | 72 (81.82%) | 55 (62.5%) | 17 (54.84%) | 1.37 (0.55-3.4) | 0.523 |
|  | Other | 47 (53.41%) | 33 (37.5%) | 14 (45.16%) |  |  |
| Education level | Primary | 14 (15.91%) | 10 (11.36%) | 4 (12.9%) | 0.86 (0.22-4.06) | 0.755 |
|  | Secondary or more | 102 (115.91%) | 76 (86.36%) | 26 (83.87%) |  |  |
| Marital status | Married | 80 (90.91%) | 56 (63.64%) | 24 (77.42%) | 0.51 (0.17-1.41) | 0.187 |
|  | Not married | 39 (44.32%) | 32 (36.36%) | 7 (22.58%) |  |  |
| Disability (mRS) | No disab | 102 (115.91%) | 74 (84.09%) | 28 (90.32%) | 0.57 (0.1-2.26) | 0.554 |
|  | Some disab | 17 (19.32%) | 14 (15.91%) | 3 (9.68%) |  |  |
| Previous stroke | No | 106 (120.45%) | 77 (87.5%) | 29 (93.55%) | 0.49 (0.05-2.43) | 0.51 |
|  | Yes | 13 (14.77%) | 11 (12.5%) | 2 (6.45%) |  |  |
| TIA | No | 96 (109.09%) | 69 (78.41%) | 27 (87.1%) | 0.54 (0.12-1.84) | 0.428 |
|  | Yes | 23 (26.14%) | 19 (21.59%) | 4 (12.9%) |  |  |
| Hypertension | No | 56 (63.64%) | 41 (46.59%) | 15 (48.39%) | 0.93 (0.38-2.3) | 1 |
|  | Yes | 63 (71.59%) | 47 (53.41%) | 16 (51.61%) |  |  |
| Diabetes | No | 102 (115.91%) | 74 (84.09%) | 28 (90.32%) | 0.57 (0.1-2.26) | 0.554 |
|  | Yes | 17 (19.32%) | 14 (15.91%) | 3 (9.68%) |  |  |
| Ischemic Heart Disease | No | 97 (110.23%) | 73 (82.95%) | 24 (77.42%) | 1.42 (0.43-4.24) | 0.591 |
|  | Yes | 22 (25%) | 15 (17.05%) | 7 (22.58%) |  |  |
| Atrial Fibrillation | No | 98 (111.36%) | 72 (81.82%) | 26 (83.87%) | 0.87 (0.23-2.81) | 1 |
|  | Yes | 21 (23.86%) | 16 (18.18%) | 5 (16.13%) |  |  |
| Ever smoker | No | 32 (36.36%) | 26 (29.55%) | 6 (19.35%) | 1.74 (0.6-5.8) | 0.349 |
|  | Yes | 87 (98.86%) | 62 (70.45%) | 25 (80.65%) |  |  |
| Lesion side | Left | 36 (40.91%) | 27 (30.68%) | 9 (29.03%) | 1.37 (0.43-4.46) | 0.605 |
|  | Right | 35 (39.77%) | 24 (27.27%) | 11 (35.48%) |  |  |
| Age | 119 \| 88 \|31 | 67.8 (15.95) | 67.85 (15.4) | 67.4 (16.85) | -0.4 (-5.7-4.8) | 0.87 |
| NIHSS (stroke severity) | 119 \| 88 \| 31 | 2 (3) | 2 (3) | 1 (2.5) | 1 (0-1) | 0.065 |
| MADRS (depression) | 116 \| 86 \| 30 | 4 (7) | 4 (7) | 3.5 (7) | 0 (-1-2) | 0.72 |
| MoCA baseline | 119 \| 88 \| 31 | 25 (6) | 26 (5) | 25 (6) | 0 (-1-2) | 0.784 |
| MoCA 3 months | 119 \| 88 \| 31 | 27 (4) | 27 (4.25) | 26 (2) | 0 (-1-2) | 0.742 |
| MoCA 12 months | 119 \| 88 \| 31 | 26 (4) | 26 (4) | 26 (4) | 0 (-1-1) | 0.956 |
| Height | 108 \| 78 \| 30 | 170 (16) | 171.5 (15) | 166 (16.75) | 5 (0-10) | 0.048 |
| Weight | 111 \| 80 \| 31 | 78 (20) | 78.5 (20.75) | 76 (17.5) | 1 (-6-8) | 0.813 |
| BMI | 104 \| 78 \| 26 | 26.91 (4.73) | 26.9 (4.27) | 26.92 (6.23) | 0.13 (-1.86-2.04) | 0.913 |
| Systolic BP | 119 \| 88 \| 31 | 140 (25.5) | 136 (25.25) | 140 (35) | -3 (-13-6) | 0.577 |
| Diastolic BP | 119 \| 88 \| 31 | 77 (13) | 77 (14) | 76 (11.5) | 0 (-4-5) | 0.713 |
| RAPA (aerobic) | 119 \| 88 \| 31 | 4 (3) | 4 (3) | 4 (2) | 1 (0-2) | 0.065 |
| RAPA (strength) | 119 \| 88 \| 31 | 0 (1) | 0 (1) | 0 (0.5) | 0 (0-0) | 0.579 |
| Charlson Cmb. Index | 119 \| 88 \| 31 | 3 (2) | 3 (2) | 3 (2) | 0 (0-0) | 0.793 |
| *p-values from Fisher exact test or Wilcoxon rank-sum tests comparing those on medication or not  **BP**=Blood pressure; **BMI**=Body mass index; **Cmb**= Comorbidity; **MADRS**=Montgomery-Åsberg Depression Rating Scale; **MoCA**=Montreal Cognitive Assessment; **mRS**=modified Rankin Scale; **NIHSS**=National Institutes of Health Stroke Scale; **RAPA**=Rapid Assessment of Physical Activity; **TIA**=Transient Ischemic Attack; **tPA**= tissue-plasminogen activator | | | | | | |
