# Supplementary figures and images for: Characterization and individual-level prediction of cognitive state in the first year after ‘mild’ stroke
Source: PLoS One. 2024 Aug 30;19(8):e0308103. doi: 10.1371/journal.pone.0308103 (PMC11364298; doi:10.1371/journal.pone.0308103)

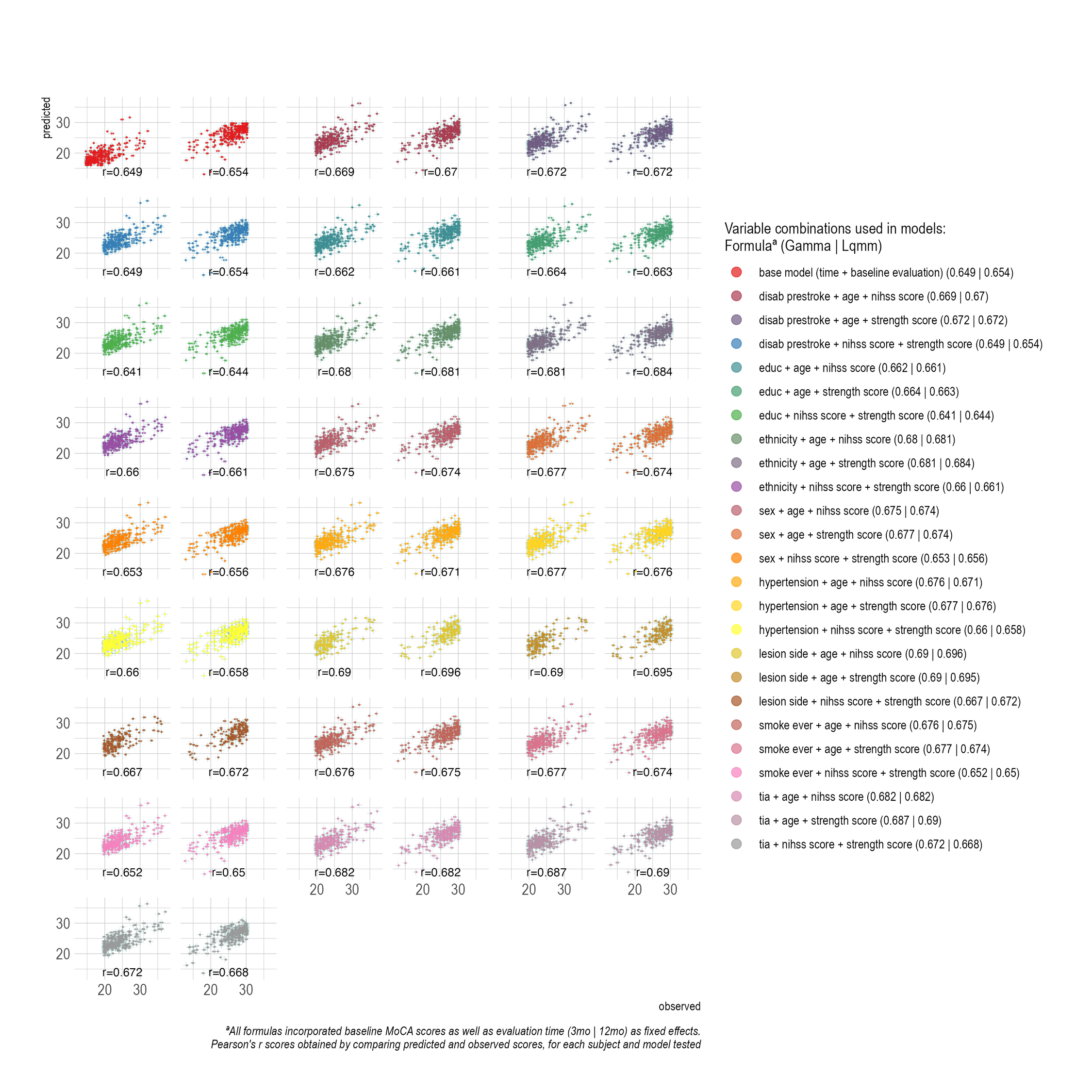

Supplement: S1 Fig — (TIF) [file pone.0308103.s007.tif]

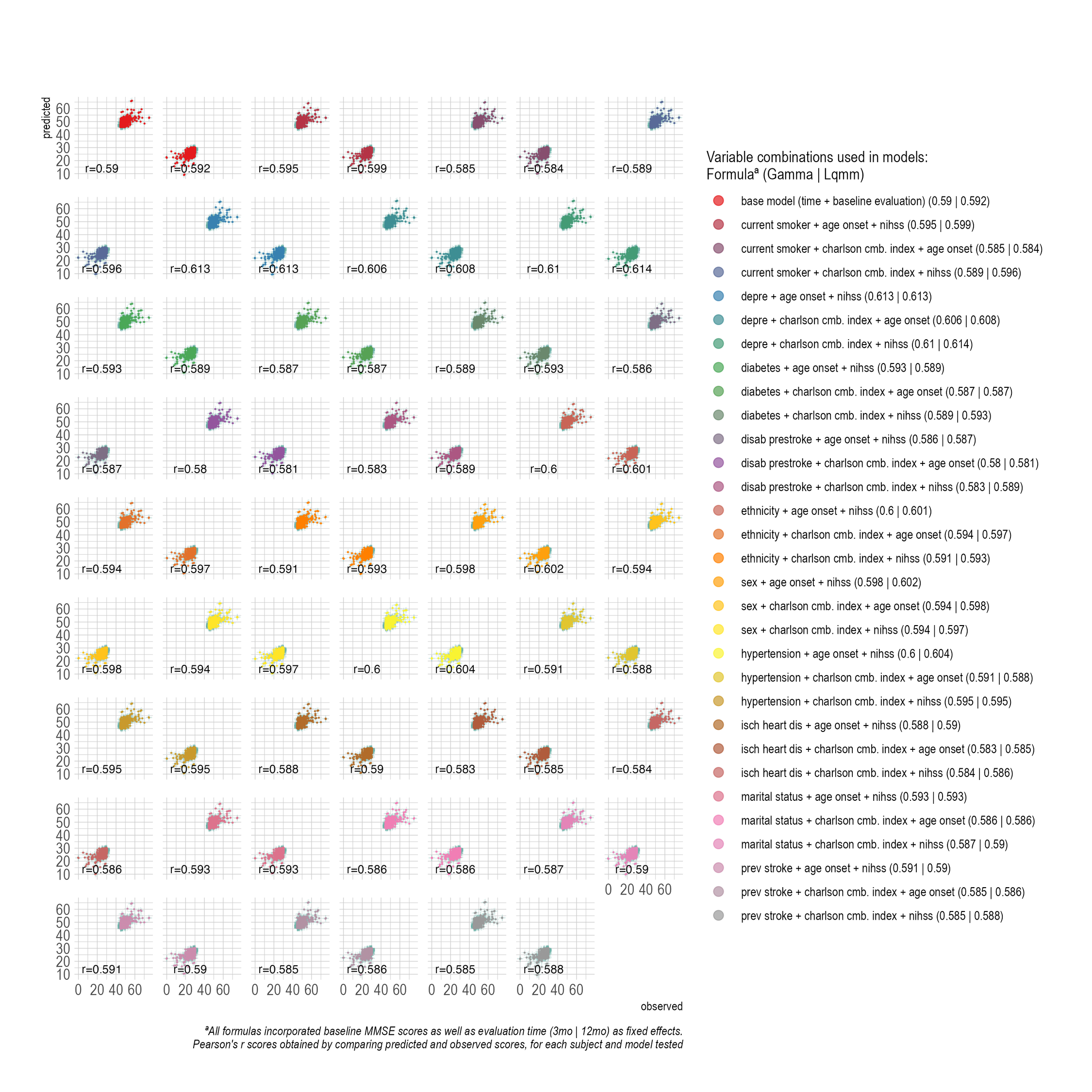

Supplement: S2 Fig — (TIF) [file pone.0308103.s008.tif]

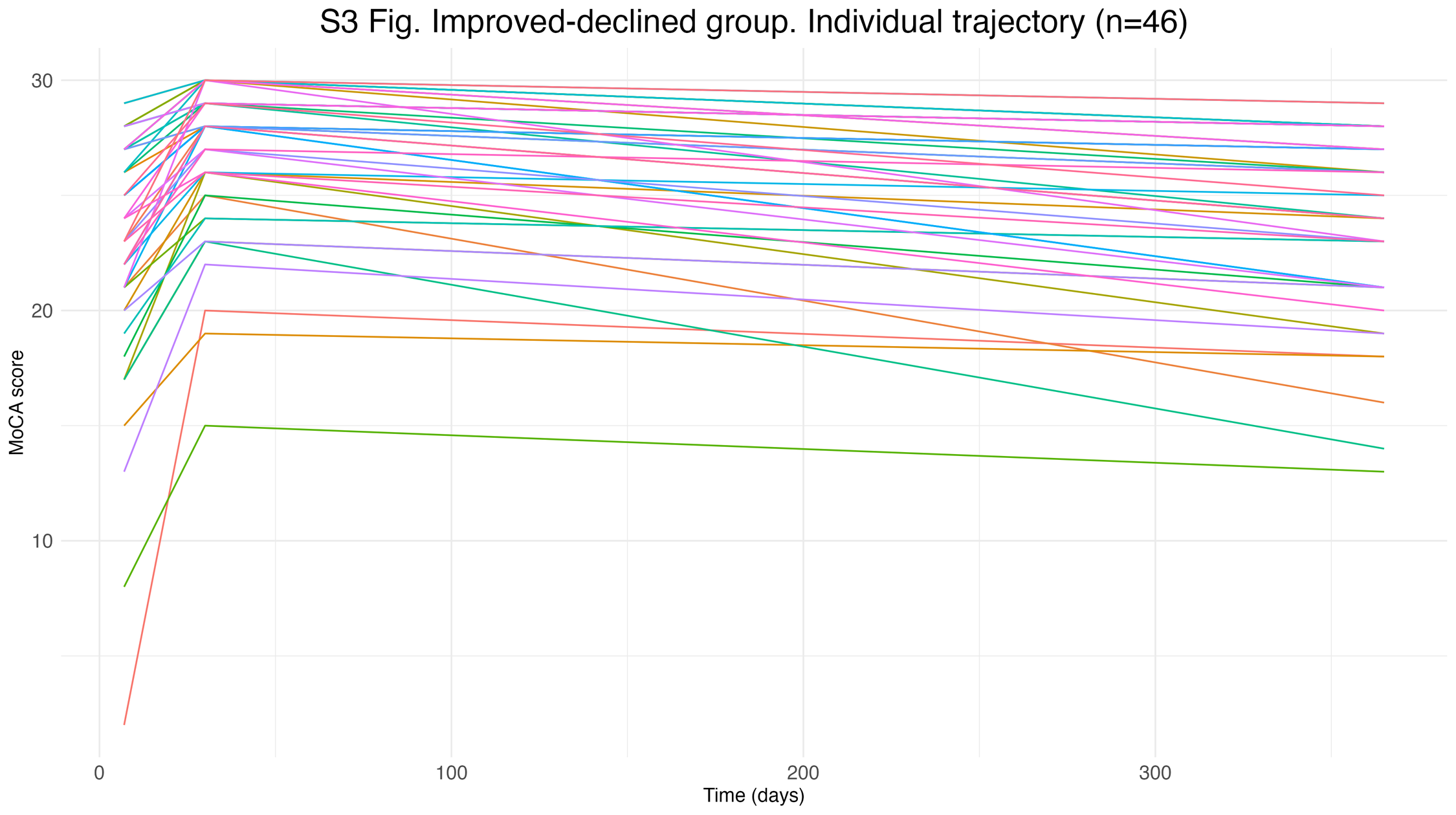

Supplement: S3 Fig — Individual trajectory of overall MoCA scores. (TIF) [file pone.0308103.s009.tif]
