# Supplementary material for: Characterization and individual-level prediction of cognitive state in the first year after ‘mild’ stroke
Source: PLoS One. 2024 Aug 30;19(8):e0308103. doi: 10.1371/journal.pone.0308103 (PMC11364298; doi:10.1371/journal.pone.0308103)

Characterization and individual-level prediction of cognitive state in the first year after ‘mild’ stroke

Supplementary Materials

Juan Pablo Saa,^1,2*^ Tamara Tse,^1^ Gerald Choon-Huat Koh,^3^ Philip Yap,^4^ Carolyn M. Baum,^5^ David E. Uribe-Rivera,^6^ Saras M. Windecker,^7^ Henry Ma,^8^ Stephen M. Davis,^9^ Geoffrey A. Donnan,^9^ and Leeanne M. Carey^1,2,10^

^1^ Occupational Therapy, School of Allied Health, Human Services and Sport, College of Science Health and Engineering, La Trobe University, Melbourne, Australia

^2^ Neurorehabilitation and Recovery, The Florey Institute of Neuroscience and Mental Health, University of Melbourne, Melbourne, Australia

^3^ Saw-Swee Hock School of Public Health, National University of Singapore, Singapore

^4^ Geriatric Medicine, Khoo Teck Puat Hospital, Singapore

^5^ School of Public Health, Washington University School of Medicine, Saint Louis, MO, USA

^6^ Commonwealth Scientific and Industrial Research Organisation (CSIRO) of Australia, Brisbane, Queensland, Australia

^7^ Telethon Kids institute, Perth, Australia

^8^ Department of Medicine, Monash Health, Monash University, Australia

^9^ Departments of Medicine and Neurology, Melbourne Brain Centre, Royal Melbourne Hospital, University of Melbourne, Melbourne, Australia

^10^ Care Economy Research Institute, La Trobe University, Bundoora, Australia

* Corresponding author

E-mail: [saajp@outlook.com](mailto:saajp@outlook.com) (JPS)

| **Table S1. Baseline characteristics of Australian and Singapore stroke cohorts for common variables across datasets** | | | | | |
| --- | --- | --- | --- | --- | --- |
| **Variables** | **Levels** | **Singapore**  **(n=191)** | **Australia**  **(n=119)** | **Estimate (95% C.I.)** | **p-value*** |
| Sex | Female | 64 (33.51%) | 37 (31.09%) | 1.12 (0.67 to 1.89) | 0.709 |
|  | Male | 127 (66.49%) | 82 (68.91%) |  |  |
| Hypertension | No | 56 (29.32%) | 56 (47.06%) | 0.47 (0.28 to 0.77) | **0.002** |
|  | Yes | 135 (70.68%) | 63 (52.94%) |  |  |
| Ischemic heart disease | No | 166 (86.91%) | 97 (81.51%) | 1.5 (0.76 to 2.95) | 0.254 |
|  | Yes | 25 (13.09%) | 22 (18.49%) |  |  |
| Diabetes | No | 120 (62.83%) | 102 (85.71%) | 0.28 (0.15 to 0.52) | **<.001** |
|  | Yes | 71 (37.17%) | 17 (14.29%) |  |  |
| Previous stroke | No | 158 (82.72%) | 106 (89.08%) | 0.59 (0.27 to 1.21) | 0.141 |
|  | Yes | 33 (17.28%) | 13 (10.92%) |  |  |
| Depression** | No | 173 (90.58%) | 92 (77.31%) | 2.87 (1.41 to 5.94) | **0.002** |
|  | Yes | 17 (8.9%) | 26 (21.85%) |  |  |
| Current smoker | No | 119 (62.3%) | 100 (84.03%) | 0.3 (0.16 to 0.55) | **<.001** |
|  | Yes | 72 (37.7%) | 18 (15.13%) |  |  |
| Ethnicity | Ethnic majority | 129 (67.54%) | 72 (60.5%) | 1.36 (0.82 to 2.25) | 0.223 |
|  | Other | 62 (32.46%) | 47 (39.5%) |  |  |
| Disability | No disability | 171 (89.53%) | 102 (85.71%) | 1.42 (0.67 to 3.01) | 0.368 |
|  | Some disab. | 20 (10.47%) | 17 (14.29%) |  |  |
| Marital status | Married | 146 (76.44%) | 80 (67.23%) | 1.58 (0.92 to 2.71) | 0.088 |
|  | Not married | 45 (23.56%) | 39 (32.77%) |  |  |
| Charlson cmb. index | Median (IQR) | 3 (1) | 3 (2) | 0 (0 to 0) | 0.351 |
| Age (years) | Median (IQR) | 59.59 (12.31) | 67.8 (15.95) | -6.635 (-9.238 to -4.006) | **<.001** |
| NIHSS (total score) | Median (IQR) | 3 (3) | 2 (3) | 1 (0 to 1) | **<.001** |
| Onset to study enrolment (days) | Median (IQR) | 0 (1) | 3.34 (2.31) | -3.07 (-3.28 to -2.84) | **<.001** |
| *p-values from Fisher and Wilcoxon rank-sum tests (as appropriate); ** Depression at admission based on Patient Health Questionnaire (PHQ-2) for Australia; and the Centers for Epidemiological Studies Depression (CES-D > 15 points) scale for Singapore; **Cmb**= Comorbidity; **IQR**=Interquartile range | | | | | |

| **Table S2. Changes in MoCA scores from baseline to 3- and 12-months post-stroke for group 3 “Improved-declined” (n=45)^*^ in START cohort** | | | | | | | | | |
| --- | --- | --- | --- | --- | --- | --- | --- | --- | --- |
| **Item (range)** | **Baseline Median (IQR)** | **3 Months Median (IQR)** | **z-val.1**  **(95% CI)** | **Change 1**  **(p-value)** | **12 Months Median (IQR)** | **z-val 2**  **(95% CI)** | **Change 2**  **(p-value)** | **z-val 3**  **(95% CI)** | **Overall change**  **(p-value)** |
| **Total score**  **(0-30 points)** | **24 (6)** | **28 (3)** | **-4 (-5, -3)** | **<.001** | **25 (6)** | **2.5 (2, 3.5)** | **<.001** | -1 (-2, 0) | **0.017** |
| **Exec/Visuosp**  **(0-5 points)** | **4 (3)** | **5 (1)** | **-1 (-1.5, -0.5)** | **<.001** | **4 (2)** | **0.5 (0.5, 1)** | **0.001** | 0 (-0.5, 0.5) | 0.715 |
| Naming  (0-3 points) | 3 (0) | 3 (0) | 0 (0, 0) | 0.173 | 3 (0) | 0 (-Inf, 0) | 0.25 | 0 (0, 0) | 0.537 |
| **Attention**  **(0-6 points)** | **10 (2)** | **10 (1)** | **-0.5 (-1, 0)** | **0.047** | 9 (2) | 0.5 (0, 0.5) | 0.146 | 0 (-1, 0) | 0.304 |
| **Language**  **(0-3 points)** | **5 (2)** | **6 (1)** | **-0.5 (-1, -0.5)** | **<.001** | **5 (2)** | **0.5 (0.5, 1)** | **<.001** | 0 (-0.5, 0.5) | 0.874 |
| **Abstraction**  **(0-2 points)** | **2 (1)** | **2 (0)** | **0 (-0.5, 0)** | **0.016** | 2 (1) | 0 (0, 0) | 0.276 | 0 (-0.5, 0) | 0.415 |
| **Delayed recall**  **(0-5 points)** | **2 (1)** | **4 (2)** | **-1.5 (-2, -1)** | **<.001** | **3 (2)** | **0.5 (0.5, 1)** | **<.001** | -1 (-1, -0.48) | **0.003** |
| **Orientation**  **(0-6 points)** | 6 (0) | 6 (0) | 0 (0, 0) | 0.062 | **6 (1)** | **0 (0, 0.5)** | **0.016** | 0 (0, 0.5) | 0.631 |
| ^*^ One patient removed due to severe aphasia at baseline.  All p-values obtained from Asymptotic Wilcoxon-Pratt signed-rank test. **Change 1**=comparison of MoCA at baseline and 3-months post-stroke; **Change 2**=comparison of MoCA at 3- and 12-months post-stroke; **Overall change**=change from baseline to 12-months.  **Exec/visuosp**=executive and visuospatial function; **IQR**=Interquartile range.  **MoCA**=Montreal Cognitive Assessment. | | | | | | | | | |

| **Table S3. Exploratory, unadjusted regression analyses predicting Montreal Cognitive Assessment (MoCA) using baseline variables in START cohort (n=119)** | | | | |
| --- | --- | --- | --- | --- |
| **Dependent variable (MoCA)** | **Time-point** | **Baseline predictor** | **Estimate (95% CI)^a^** | **p-value^b^** |
|  |  |  | **Bivariate binary logistic regression** | |
| Cognitive impairment  (MoCA < 24) | Baseline (day 3-7) | Secondary education or more | 0.125 (0.033 to 0.478) | **0.002** |
|  | 3 months | Ethnicity (other) | 10 (1.940 to 51.542) | **0.006** |
|  |  | Ever smoking (yes) | 8.662 (1.109 to 67.638) | **0.04** |
|  |  | MoCA score | 0.688 (0.551 to 0.858) | **0.001** |
|  | 12 months | Ethnicity (other) | 8.437 (1.624 to 43.843) | **0.011** |
|  |  | Ever smoking (yes) | -2 (-3.8 to -0.2) | **0.031** |
|  |  | MoCA score | 0.561 (0.397 to 0.795) | **0.001** |
|  |  |  | **Bivariate quantile regression** | |
| MoCA median score | Baseline (day 3-7) | Secondary education or more | 5 (2.709 to 7.291) | **<.001** |
|  |  | Some disability (mRS 1–2 points) | -3 (-5.787 to -0.213) | **0.037** |
|  | 3 months | Secondary education or more | 2 (0.367 to 3.633) | **0.018** |
|  |  | RAPA strength score | 0.667 (0.065 to 1.268) | **0.032** |
|  |  | MoCA score | 0.5 (0.366 to 0.634) | **<.001** |
|  | 12 months | Secondary education or more | 3.176 (0.146 to 6.207) | **0.042** |
|  |  | Age | -0.078 (-0.126 to -0.029) | **0.002** |
|  |  | RAPA strength score | 1 (0.155 to 1.845) | **0.022** |
|  |  | MoCA score | 0.587 (0.382 to 0.791) | **<.001** |
|  |  |  | **Bivariate mixed quantile regression** | |
| MoCA median score | Longitudinal analysis | Secondary education or more | 4 (1.423 to 6.577) | **0.003** |
|  |  | Ischemic heart disease | -2 (-3.946 to -0.054) | **0.044** |
|  |  | Age | -0.087 (-0.134 to -0.04) | **0.001** |
|  |  | RAPA strength score | 0.667 (0.090 to 1.244) | **0.024** |
|  |  |  | **Bivariate mixed Gamma regression** | |
| MoCA Gamma score | Longitudinal analysis | Age | 1.174 (0.3 to 2.048) | **0.008** |
|  |  | NIHSS score | 0.986 (0.132 to 1.839) | **0.024** |
|  |  | RAPA strength score | -0.94 (-1.844 to -0.036) | **0.042** |
| **mRS**=modified Rankin Scale; **RAPA**=Rapid Assessment of Physical Activity; **NIHSS**=National Institutes of Health Stroke Scale; **MoCA**=Montreal Cognitive Assessment  **Note:** Variables tested in all model formulations included sex, dichotomized education (less than secondary / secondary or more), previous stroke, previous TIA, hypertension, atrial fibrillation, diabetes, ischemic heart disease, pre-morbid disability (mRS), ethnicity (Australia/NZ or other), age, stroke severity (NIHSS), depression (MADRS), aerobic and strength capacity (RAPA), and body mass index (kg/m^2^). *Estimates represent slope of linear relationships (quantile regression and quantile mixed regression), and odds ratio, (binary logistic regression). | | | | |

| **Table S4. Trajectory profiles of stroke clusters at 12-months post-stroke in START cohort, based on changes in MoCA scores** | | | | | | | | | | |
| --- | --- | --- | --- | --- | --- | --- | --- | --- | --- | --- |
| **Variable*** | **1.overall improver** | **2.improved-stable** | **3.improved-declined** | **4.stable-improved** | **5.overall stable** | **6.stable-declined** | **7.declined-improved** | **8.declined-stable** | **9.overall decliner** |  |
| **n (%)** | 22 (18.49%) | 15 (12.61%) | 46 (38.66%) | 2 (1.68%) | 2 (1.68%) | 7 (5.88%) | 12 (10.08%) | 6 (5.04%) | 7 (5.88%) |  |
| **Males (%)** | 16 (13.45%) | 8 (6.72%) | 32 (26.89%) | 2 (1.68%) | 1 (0.84%) | 6 (5.04%) | 10 (8.4%) | 3 (2.52%) | 4 (3.36%) |  |
| **High-school or more (%)** | 15 (12.61%) | 14 (11.76%) | 39 (32.77%) | 2 (1.68%) | 2 (1.68%) | 7 (5.88%) | 11 (9.24%) | 5 (4.2%) | 7 (5.88%) |  |
| **Marital status (%)** | 15 (12.61%) | 13 (10.92%) | 31 (26.05%) | 0 (0%) | 1 (0.84%) | 5 (4.2%) | 8 (6.72%) | 3 (2.52%) | 4 (3.36%) |  |
| **Disability pre-stroke (%)** | 3 (2.52%) | 1 (0.84%) | 9 (7.56%) | 0 (0%) | 0 (0%) | 1 (0.84%) | 0 (0%) | 3 (2.52%) | 0 (0%) |  |
| **Age (years)** | 22, 66 (13.22) | 15, 62.8 (20.7) | 46, 68.3 (13.35) | 2, 75.05 (6.05) | 2, 61.3 (18.6) | 7, 70.3 (20.45) | 12, 66.05 (14.5) | 6, 72.1 (13.75) | 7, 69.9 (20.05) |  |
| **CharIson Cmb. Index** | 22, 1.5 (2) | 15, 1 (2) | 46, 3 (2) | 2, 2 (1) | 2, 3 (0) | 7, 3 (1) | 12, 3 (2) | 6, 3 (1.5) | 7, 3 (0) |  |
| **NIHSS score w1** | 22, 141.5 (29.75) | 15, 132 (25) | 46, 135 (26.5) | 2, 151.5 (25.5) | 2, 140 (10) | 7, 160 (17) | 12, 141.5 (31) | 6, 145 (20.5) | 7, 136 (13) |  |
| **NIHSS score 3mth** | 22, 77.5 (20) | 15, 75 (15.5) | 46, 75 (10) | 2, 74 (3) | 2, 80 (0) | 7, 85 (13.5) | 12, 79 (7) | 6, 75.5 (11) | 7, 77 (7.5) |  |
| **NIHSS score 12mth** | 22, 128 (13.25) | 15, 122 (20) | 45, 130 (20) | 2, 157 (16) | 2, 100 (5) | 7, 126 (12.5) | 12, 122 (30.5) | 6, 138 (12.25) | 7, 115 (10) |  |
| **Systolic bp w1** | 22, 75.5 (15) | 15, 75 (14.5) | 45, 76 (10) | 2, 89 (4) | 2, 55 (5) | 7, 65 (10.5) | 12, 74 (18.75) | 6, 76 (11) | 7, 68 (9.5) |  |
| **Diastolic bp w1** | 22, 126.5 (18.5) | 15, 125 (13.5) | 46, 125 (14.5) | 2, 147 (23) | 2, 120 (10) | 7, 122 (17) | 12, 124.5 (12.5) | 6, 137 (26) | 7, 120 (4.5) |  |
| **Systolic bp mo3** | 22, 66.5 (20.25) | 15, 73 (10) | 46, 72.5 (12.75) | 2, 64.5 (4.5) | 2, 65 (5) | 7, 70 (7.5) | 12, 75.5 (12.25) | 6, 85 (22) | 7, 67 (5) |  |
| **Diastolic bp mo3** | 22, 22 (3) | 15, 27 (1.5) | 46, 23.5 (6) | 2, 28.5 (0.5) | 2, 27 (3) | 7, 30 (6) | 12, 26.5 (4.25) | 6, 28 (2.25) | 7, 28 (3) |  |
| **Systolic bp 12mth** | 22, 25 (1.75) | 15, 29 (2) | 46, 28 (3) | 2, 28.5 (0.5) | 2, 27 (3) | 7, 30 (6) | 12, 24 (3.25) | 6, 25.5 (4.5) | 7, 26 (1.5) |  |
| **Diastolic bp 12mth** | 22, 27.5 (2.75) | 15, 29 (2) | 46, 24.5 (6) | 2, 30 (0) | 2, 27 (3) | 7, 26 (9.5) | 12, 26 (2.25) | 6, 25.5 (4.5) | 7, 24 (4) |  |
| **RAPA aerobic score w1** | 17, 27 (2) | 14, 28.5 (2.75) | 28, 27.5 (2.25) | 2, 27 (1) |  | 6, 27 (3.75) | 7, 27 (4) | 3, 28 (2.5) | 4, 27 (3.5) |  |
| **RAPA strength score w1** | 17, 28 (1) | 14, 29 (1.75) | 26, 27 (3) | 2, 29 (1) |  | 6, 27.5 (4.75) | 7, 25 (4.5) | 3, 29 (1) | 4, 25 (3.25) |  |
| **RAPA aerobic score 3mth** | 17, 2.4 (0.8) | 14, 2.25 (1.1) | 28, 2.25 (0.92) | 2, 2.65 (0.15) |  | 6, 2.05 (0.65) | 7, 2 (0.45) | 3, 2.2 (0.75) | 4, 1.8 (0.38) |  |
| **RAPA strength score 3mth** | 17, 2.3 (1.2) | 14, 1.95 (0.52) | 26, 2.4 (1.1) | 2, 2.05 (0.05) |  | 6, 1.9 (0.9) | 7, 2.3 (1.03) | 3, 2.1 (0.55) | 4, 1.8 (0.38) |  |
| **RAPA aerobic score 12mth** | 17, 29 (8) | 14, 33 (5.25) | 28, 29 (6) | 2, 28 (7) |  | 6, 31 (9.75) | 7, 27 (4) | 3, 29 (4) | 4, 28 (7.75) |  |
| **RAPA strength score 12mth** | 17, 29 (9) | 14, 32.5 (6) | 26, 27.5 (8.75) | 2, 31.5 (3.5) |  | 6, 31 (11.5) | 7, 29 (6) | 3, 32 (3) | 4, 27 (7.5) |  |
| **MADRS score w1** | 17, 153 (146) | 14, 89 (65.5) | 28, 103 (145.25) | 2, 127.5 (65.5) |  | 6, 85.5 (180) | 7, 77 (65) | 3, 92 (29) | 4, 97 (103.75) |  |
| **MADRS score 3mth** | 17, 120 (163) | 14, 91 (51.5) | 26, 89 (97.5) | 2, 87.5 (40.5) |  | 6, 85 (172.5) | 7, 98 (38) | 3, 62 (31) | 4, 106.5 (97.75) |  |
| **MADRS score 12mth** | 17, 9 (2) | 14, 9.5 (1.75) | 28, 8.5 (2.5) | 2, 9 (0) |  | 6, 11 (1.5) | 7, 10 (3) | 3, 8 (4) | 4, 9 (0.5) |  |
| **MoCA score w1** | 17, 9 (4) | 14, 10 (2) | 26, 8 (1.75) | 2, 10 (0) |  | 6, 11 (1.5) | 7, 10 (3) | 3, 10 (4) | 4, 8.5 (2) |  |
| **MoCA score 3mth** | 17, 7 (1) | 14, 8 (2.75) | 28, 7 (2.25) | 2, 6.5 (0.5) |  | 6, 8 (5.5) | 7, 6 (2.5) | 3, 6 (4) | 3, 7 (3) |  |
| **MoCA score 12mth** | 17, 7 (1) | 14, 8 (1.75) | 26, 7 (2) | 2, 6.5 (0.5) |  | 6, 7.5 (4.75) | 7, 7 (0) | 3, 7 (3.5) | 4, 8 (3) |  |
| **MMSE score 3mth** | 22, 5 (7) | 15, 6 (6) | 44, 2.5 (5.5) | 2, 4 (1) | 2, 11 (5) | 7, 1 (2.5) | 11, 8 (10) | 6, 7 (7.5) | 7, 3 (13.5) |  |
| **MMSE score 12mth** | 22, 3.5 (6.5) | 15, 4 (9.5) | 46, 3.5 (10.5) | 2, 0.5 (0.5) | 2, 12 (0) | 7, 4 (5.5) | 12, 6.5 (9.5) | 6, 4 (0.75) | 7, 4 (5) |  |
| **Stroop ratio 3mth** | 22, 3 (5) | 15, 2 (6) | 43, 6 (12.5) | 2, 0.5 (0.5) | 2, 12 (3) | 7, 5 (5.5) | 12, 4.5 (7) | 6, 2.5 (2.5) | 7, 6 (5) |  |
| **Stroop ratio 12mth** | 22, 2 (2) | 15, 1 (1.5) | 46, 2 (3) | 2, 0.5 (0.5) | 2, 4 (2) | 7, 0 (3.5) | 12, 1 (3) | 6, 0.5 (1) | 7, 1 (3) |  |
| **Ravens score 3mth** | 22, 1 (1) | 15, 0 (1) | 46, 0 (1) | 2, 0 (0) | 2, 0 (0) | 7, 0 (0.5) | 12, 0 (1.25) | 6, 1 (0.75) | 7, 1 (1) |  |
| **Ravens score 12mth** | 22, 0 (1) | 15, 0 (0.5) | 46, 0 (1) | 2, 0 (0) | 2, 0.5 (0.5) | 7, 0 (1) | 12, 0 (1) | 6, 0.5 (1) | 7, 0 (1.5) |  |
| **TMT-B time taken 3mth** | 22, 100 (0) | 15, 100 (0) | 46, 100 (5) | 2, 100 (0) | 2, 100 (0) | 7, 100 (0) | 12, 100 (0) | 6, 100 (0) | 7, 100 (2.5) |  |
| **TMT-B time taken 12mth** | 22, 100 (0) | 15, 100 (0) | 46, 100 (3.75) | 2, 100 (0) | 2, 100 (0) | 7, 100 (0) | 12, 100 (0) | 6, 100 (3.75) | 7, 100 (0) |  |
| **Digit span forward 3mth** | 22, 1 (0.75) | 15, 1 (2) | 46, 1 (1) | 2, 0.5 (0.5) | 2, 1 (0) | 7, 1 (1) | 12, 1 (1) | 6, 1 (0.75) | 7, 1 (0.5) |  |
| **Digit span forward 12mth** | 22, 1 (1) | 15, 0 (1) | 46, 1 (2) | 2, 0 (0) | 2, 1 (0) | 7, 1 (1) | 12, 1 (1) | 6, 1.5 (1.75) | 7, 1 (1) |  |
| **Digit span backward 3mth** | 22, 4 (3) | 15, 3 (1) | 46, 4 (3) | 2, 5 (2) | 2, 5.5 (1.5) | 7, 2 (2.5) | 12, 5.5 (2) | 6, 3.5 (1) | 7, 4 (2) |  |
| **Digit span backward 12mth** | 22, 0 (0.75) | 15, 0 (1.5) | 46, 0 (0.75) | 2, 1.5 (1.5) | 2, 3 (0) | 7, 0 (0) | 12, 0.5 (2) | 6, 0 (0) | 7, 0 (1) |  |
| **Barthel score 3mth** | 22, 5.5 (3.5) | 15, 4 (2) | 45, 4 (3) | 2, 6 (0) | 2, 4.5 (1.5) | 7, 4 (1.5) | 12, 6 (2) | 6, 4 (2.25) | 7, 4 (3) |  |
| **Barthel score 12mth** | 22, 1 (2) | 15, 0 (1) | 45, 0 (1) | 2, 3 (0) | 2, 1.5 (1.5) | 7, 0 (1) | 12, 1 (2) | 6, 1 (2) | 7, 1 (3) |  |
| **mRS score 3mth** | 22, 4 (3.75) | 15, 4 (3) | 46, 3.5 (4) | 2, 6 (1) | 2, 3.5 (0.5) | 7, 3 (2.5) | 12, 4 (3) | 6, 3 (1.5) | 7, 3 (0.5) |  |
| **mRS score 12mth** | 22, 0 (1.75) | 15, 1 (2) | 46, 0 (1) | 2, 1.5 (1.5) | 2, 0.5 (0.5) | 7, 0 (0) | 12, 0 (3) | 6, 0 (0) | 7, 0 (0.5) |  |
| **ACS (RALN) 3mth** | 17, 98 (6) | 14, 96.5 (7.75) | 28, 97 (25.5) | 2, 100 (0) |  | 6, 97 (8) | 7, 89 (22.5) | 3, 94 (11.5) | 4, 93 (12.5) |  |
| **ACS (RALN) 12mth** | 17, 98 (4) | 14, 100 (3.75) | 26, 98 (8.5) | 2, 100 (0) |  | 6, 96 (20.25) | 7, 100 (4) | 3, 100 (8) | 4, 97.5 (9.75) |  |
| **WSAS score 3mth** | 22, 3.5 (8.62) | 15, 8 (10.25) | 45, 2 (11) | 2, 0 (0) | 2, 14.5 (13.5) | 7, 0.5 (8.5) | 12, 2 (5.5) | 6, 4 (4.75) | 7, 12 (15) |  |
| **WSAS score 12mth** | 22, 2 (5.5) | 15, 0 (5.5) | 46, 3.5 (12.5) | 2, 0 (0) | 2, 12.5 (6.5) | 7, 0 (5) | 12, 0 (8.25) | 6, 3.75 (3.38) | 7, 0 (8.5) |  |
| **SIS total 3mo** | 22, 751.87 (67.23) | 14, 746.87 (89.56) | 45, 728.42 (128.64) | 2, 794.44 (2.78) | 2, 688.2 (9.44) | 7, 766.67 (67.86) | 12, 745.24 (66.06) | 6, 719.36 (32.78) | 7, 755.44 (48.53) |  |
| **SIS total 12mo** | 22, 772.22 (60.04) | 15, 766.67 (48.18) | 45, 736.31 (142.56) | 2, 794.44 (5.56) | 2, 631.31 (89.16) | 7, 750.64 (57.68) | 12, 756.85 (70.4) | 6, 681.25 (60.71) | 7, 746.03 (33.17) |  |
| *All continuous variables are reported as **count, median (IQR)**  **ACS**=Activity Card Sort – Retained Activity Level; **BP**=blood pressure; **MADRS**=Montgomery-Åsberg Depression Rating Scale; **MMSE**=Mini-Mental State Examination; **MoCA**=Montreal Cognitive Assessment; **mRS**=Modified Rankin Scale; **NIHSS**=National Institutes of Health Stroke Scale; **RAPA**=Rapid Assessment of Physical Activity; **SIS**=Stroke Impact Scale; **TMT**=Trail Making Test; **w1**= baseline; **WSAS**=Work and Social Adjustment Scale. | | | | | | | | | | |

| **Table S5. Post-hoc comparisons of stroke clusters in START cohort based on MoCA scores** | | | | | | | | | | |
| --- | --- | --- | --- | --- | --- | --- | --- | --- | --- | --- |
| **Variables** | **1.overall improver** | **3.improved-declined** | **z-value 1** | **95% CI 1** | **p-value 1** | **9.overall decliner** | **z-value 2** | **95% CI 2** | **p-value 2** |  |
| **n (%)** | 22 (18.49%) | 46 (38.66%) |  |  |  | 7 (5.88%) |  |  |  |  |
| **Males (%)** | 16 (13.45%) | 32 (26.89%) | 0.859 | 0.226, 2.969 | 1 | 4 (3.36%) | 0.513 | 0.063, 4.56 | 0.642 |  |
| **High-school or more (%)** | 15 (12.61%) | 39 (32.77%) | 2.559 | 0.584, 11.317 | 0.175 | 7 (5.88%) | Inf | 0.406, Inf | 0.288 |  |
| **Marital status, married (%)** | 15 (12.61%) | 31 (26.05%) | 1.036 | 0.312, 3.672 | 1 | 4 (3.36%) | 1.58 | 0.181, 12.466 | 0.665 |  |
| **Pre-stroke disability (%)** | 3 (2.52%) | 9 (7.56%) | 1.531 | 0.329, 9.815 | 0.738 | 0 (0%) | 0 | 0, 7.977 | 0.557 |  |
| **Age** | 22, 66 (13.22) | 46, 68.3 (13.35) | -0.714 | -8.3, 3.7 | 0.48 | 7, 69.9 (20.05) | -0.051 | -10.4, 17.8 | 0.97 |  |
| **Charlson cmb. index** | 22, 1.5 (2) | 46, 3 (2) | -1.269 | -2, 0 | 0.207 | 7, 3 (0) | -1.928 | -2, 0 | 0.058 |  |
| **NIHSS score w1** | 22, 2 (2) | 46, 2 (3) | 0.743 | -1, 1 | 0.462 | 7, 1 (3) | 0.233 | -2, 2 | 0.821 |  |
| **NIHSS score 3mth** | 22, 1 (1) | 46, 0 (1) | 1.757 | 0, 1 | 0.078 | 7, 1 (1) | -0.383 | -1, 1 | 0.763 |  |
| **NIHSS score 12mth** | 22, 0 (1) | 46, 0 (1) | -0.095 | 0, 0 | 0.921 | 7, 0 (1.5) | -0.543 | -1, 0 | 0.59 |  |
| **Systolic bp w1** | 22, 141.5 (29.75) | 46, 135 (26.5) | 1.266 | -5, 17 | 0.208 | 7, 136 (13) | 0.204 | -16, 20 | 0.852 |  |
| **Diastolic bp w1** | 22, 77.5 (20) | 46, 75 (10) | 1.642 | 0, 12 | 0.102 | 7, 77 (7.5) | 0.818 | -5, 18 | 0.43 |  |
| **Systolic bp mo3** | 22, 128 (13.25) | 45, 130 (20) | 0.482 | -5, 10 | 0.635 | 7, 115 (10) | 2.194 | 2, 20 | **0.027** |  |
| **Diastolic bp mo3** | 22, 75.5 (15) | 45, 76 (10) | 0.925 | -4, 9 | 0.36 | 7, 68 (9.5) | 1.839 | 0, 19 | 0.067 |  |
| **Systolic bp 12mth** | 22, 126.5 (18.5) | 46, 125 (14.5) | 0.493 | -5, 8 | 0.627 | 7, 120 (4.5) | 0.921 | -5, 15 | 0.373 |  |
| **Diastolic bp 12mth** | 22, 66.5 (20.25) | 46, 72.5 (12.75) | -1.558 | -11, 1 | 0.121 | 7, 67 (5) | -0.077 | -9, 12 | 0.95 |  |
| **RAPA aerobic score w1** | 22, 4 (3) | 46, 4 (3) | -0.027 | -1, 1 | 0.981 | 7, 4 (2) | 0.209 | -2, 2 | 0.851 |  |
| **RAPA strength score w1** | 22, 0 (0.75) | 46, 0 (0.75) | 0.254 | 0, 0 | 0.809 | 7, 0 (1) | -0.13 | 0, 0 | 0.883 |  |
| **RAPA aerobic score 3mth** | 22, 5.5 (3.5) | 45, 4 (3) | 1.571 | 0, 2 | 0.117 | 7, 4 (3) | 0.655 | -1, 2 | 0.544 |  |
| **RAPA strength score 3mth** | 22, 1 (2) | 45, 0 (1) | 1.151 | 0, 1 | 0.261 | 7, 1 (3) | -0.568 | -2, 1 | 0.583 |  |
| **RAPA aerobic score 12mth** | 22, 4 (3.75) | 46, 3.5 (4) | 0.742 | -1, 1 | 0.463 | 7, 3 (0.5) | 0.674 | -1, 3 | 0.519 |  |
| **RAPA strength score 12mth** | 22, 0 (1.75) | 46, 0 (1) | -0.44 | 0, 0 | 0.666 | 7, 0 (0.5) | 0.422 | 0, 1 | 0.714 |  |
| **MADRS score w1** | 22, 5 (7) | 44, 2.5 (5.5) | 0.92 | -1, 4 | 0.362 | 7, 3 (13.5) | -0.437 | -10, 4 | 0.681 |  |
| **MADRS score 3mth** | 22, 3.5 (6.5) | 46, 3.5 (10.5) | -0.438 | -3, 2 | 0.666 | 7, 4 (5) | -0.592 | -4, 2 | 0.571 |  |
| **MADRS score 12mth** | 22, 3 (5) | 43, 6 (12.5) | -1.845 | -7, 0 | 0.065 | 7, 6 (5) | -1.507 | -6, 1 | 0.14 |  |
| **MoCA score w1** | 22, 22 (3) | 46, 23.5 (6) | -1.513 | -4, 0 | 0.132 | 7, 28 (3) | -3.591 | -8, -3 | **0.001** |  |
| **MoCA score 3mth** | 22, 25 (1.75) | 46, 28 (3) | -2.885 | -3, -1 | **0.003** | 7, 26 (1.5) | -0.572 | -2, 1 | 0.584 |  |
| **MoCA score 12mth** | 22, 27.5 (2.75) | 46, 24.5 (6) | 3.28 | 1, 5 | **0.001** | 7, 24 (4) | 2.547 | 1, 7 | **0.009** |  |
| **MMSE score 3mth** | 17, 27 (2) | 28, 27.5 (2.25) | -0.57 | -2, 1 | 0.577 | 4, 27 (3.5) | 0.597 | -1, 6 | 0.609 |  |
| **MMSE score 12mth** | 17, 28 (1) | 26, 27 (3) | 0.731 | -1, 2 | 0.474 | 4, 25 (3.25) | 1.318 | -1, 5 | 0.207 |  |
| **Stroop ratio 3mth** | 17, 2.4 (0.8) | 28, 2.25 (0.92) | 0.517 | -0.3, 0.6 | 0.613 | 4, 1.8 (0.38) | 1.973 | 0, 1.6 | **0.048** |  |
| **Stroop ratio 12mth** | 17, 2.3 (1.2) | 26, 2.4 (1.1) | 0.934 | -0.3, 0.6 | 0.358 | 4, 1.8 (0.38) | 1.709 | -0.1, 1.5 | 0.095 |  |
| **Ravens score 3mth** | 17, 29 (8) | 28, 29 (6) | -0.164 | -3, 3 | 0.875 | 4, 28 (7.75) | 0 | -7, 8 | 1 |  |
| **Ravens score 12mth** | 17, 29 (9) | 26, 27.5 (8.75) | -0.1 | -3, 3 | 0.926 | 4, 27 (7.5) | -0.18 | -7, 7 | 0.88 |  |
| **TMT-B time taken 3mth** | 17, 153 (146) | 28, 103 (145.25) | 1.78 | -1, 85 | 0.076 | 4, 97 (103.75) | 1.03 | -108, 167 | 0.324 |  |
| **TMT-B time taken 12mth** | 17, 120 (163) | 26, 89 (97.5) | 0.857 | -23, 62 | 0.399 | 4, 106.5 (97.75) | 0.314 | -97, 146 | 0.782 |  |
| **Digit span forward 3mth** | 17, 9 (2) | 28, 8.5 (2.5) | 0.486 | -1, 2 | 0.634 | 4, 9 (0.5) | 0.275 | -1, 2 | 0.789 |  |
| **Digit span forward 12mth** | 17, 9 (4) | 26, 8 (1.75) | 0.825 | -1, 2 | 0.42 | 4, 8.5 (2) | 0.412 | -2, 3 | 0.713 |  |
| **Digit span backward 3mth** | 17, 7 (1) | 28, 7 (2.25) | 0.764 | -1, 1 | 0.452 | 3, 7 (3) | -0.275 | -5, 2 | 0.869 |  |
| **Digit span backward 12mth** | 17, 7 (1) | 26, 7 (2) | 0.013 | -1, 1 | 0.995 | 4, 8 (3) | -0.412 | -3, 2 | 0.715 |  |
| **Barthel score 3mth** | 22, 100 (0) | 46, 100 (5) | 0.865 | 0, 0 | 0.405 | 7, 100 (2.5) | 0.433 | 0, 0 | 0.871 |  |
| **Barthel score 12mth** | 22, 100 (0) | 46, 100 (3.75) | 0.706 | 0, 0 | 0.491 | 7, 100 (0) | -0.349 | 0, 0 | 0.741 |  |
| **mRS score 3mth** | 22, 1 (0.75) | 46, 1 (1) | 0.662 | 0, 1 | 0.513 | 7, 1 (0.5) | -0.307 | -1, 1 | 0.841 |  |
| **mRS score 12mth** | 22, 1 (1) | 46, 1 (2) | -1.656 | -1, 0 | 0.101 | 7, 1 (1) | 0.113 | -1, 1 | 1 |  |
| **ACS (RALN) 3mth** | 17, 98 (6) | 28, 97 (25.5) | 1.988 | 0, 20 | **0.047** | 4, 93 (12.5) | 1.084 | -4, 20 | 0.303 |  |
| **ACS (RALN) 12mth** | 17, 98 (4) | 26, 98 (8.5) | -0.352 | -3, 3 | 0.733 | 4, 97.5 (9.75) | -0.272 | -7, 15 | 0.825 |  |
| **WSAS score 3mth** | 22, 3.5 (8.62) | 45, 2 (11) | 0.048 | -3, 2 | 0.965 | 7, 12 (15) | -0.521 | -12, 4 | 0.62 |  |
| **WSAS score 12mth** | 22, 2 (5.5) | 46, 3.5 (12.5) | -1.61 | -4.5, 0 | 0.109 | 7, 0 (8.5) | -0.163 | -7, 2 | 0.884 |  |
| **SIS total 3mo** | 22, 751.87 (67.23) | 45, 728.42 (128.64) | 0.848 | -20.635, 51.875 | 0.401 | 7, 755.44 (48.53) | -0.051 | -45.764, 44.563 | 0.972 |  |
| **SIS total 12mo** | 22, 772.22 (60.04) | 45, 736.31 (142.56) | 1.549 | -4.067, 66.855 | 0.123 | 7, 746.03 (33.17) | 0.892 | -19.305, 49.891 | 0.386 |  |
| *All continuous variables are reported as **count, median (IQR).**  **ACS (RALN)**=Activity Card Sort – Retained Activity Level (including new activities); **MADRS**=Montgomery-Åsberg Depression Rating Scale; **MMSE**=Mini-Mental State Examination; **MoCA**=Montreal Cognitive Assessment; **NIHSS**=National Institutes of Health Stroke Scale; **RAPA**=Rapid Assessment of Physical Activity; **SIS**=Stroke Impact Scale; **TMT-B**=Trail Making Test-Part B; **w1**= baseline; **WSAS**=Work and Social Adjustment Scale; z-value 1 and p-value 1 indicate statistical differences between cluster 1 (overall-improvers) and cluster 3 (improved-declined); z-value 2 and p-value 2 indicate statistical differences between cluster 1 (overall-improvers) and cluster 9 (overall decliners); | | | | | | | | | | |

| **Table S6. Comparison of START participants on antiplatelet, tPA, or anticoagulant medication (n=31) versus those not on those medications (n=88)** | | | | | | |
| --- | --- | --- | --- | --- | --- | --- |
| **Variables** | **Categories & sample size All \| No med \| On meds** | **All** | **No tPA, anticoagulant or antiplatelet medication (n=88)** | **On tPA, anticoagulant or antiplatelet medication (n=31)** | **Estimate (95% C.I.)** | **p-value** |
| Sex | Female | 37 (42.05%) | 26 (29.55%) | 11 (35.48%) | 0.76 (0.3-2.03) | 0.652 |
|  | Male | 82 (93.18%) | 62 (70.45%) | 20 (64.52%) |  |  |
| Ethnicity | Australian/NZ | 72 (81.82%) | 55 (62.5%) | 17 (54.84%) | 1.37 (0.55-3.4) | 0.523 |
|  | Other | 47 (53.41%) | 33 (37.5%) | 14 (45.16%) |  |  |
| Education level | Primary | 14 (15.91%) | 10 (11.36%) | 4 (12.9%) | 0.86 (0.22-4.06) | 0.755 |
|  | Secondary or more | 102 (115.91%) | 76 (86.36%) | 26 (83.87%) |  |  |
| Marital status | Married | 80 (90.91%) | 56 (63.64%) | 24 (77.42%) | 0.51 (0.17-1.41) | 0.187 |
|  | Not married | 39 (44.32%) | 32 (36.36%) | 7 (22.58%) |  |  |
| Disability (mRS) | No disab | 102 (115.91%) | 74 (84.09%) | 28 (90.32%) | 0.57 (0.1-2.26) | 0.554 |
|  | Some disab | 17 (19.32%) | 14 (15.91%) | 3 (9.68%) |  |  |
| Previous stroke | No | 106 (120.45%) | 77 (87.5%) | 29 (93.55%) | 0.49 (0.05-2.43) | 0.51 |
|  | Yes | 13 (14.77%) | 11 (12.5%) | 2 (6.45%) |  |  |
| TIA | No | 96 (109.09%) | 69 (78.41%) | 27 (87.1%) | 0.54 (0.12-1.84) | 0.428 |
|  | Yes | 23 (26.14%) | 19 (21.59%) | 4 (12.9%) |  |  |
| Hypertension | No | 56 (63.64%) | 41 (46.59%) | 15 (48.39%) | 0.93 (0.38-2.3) | 1 |
|  | Yes | 63 (71.59%) | 47 (53.41%) | 16 (51.61%) |  |  |
| Diabetes | No | 102 (115.91%) | 74 (84.09%) | 28 (90.32%) | 0.57 (0.1-2.26) | 0.554 |
|  | Yes | 17 (19.32%) | 14 (15.91%) | 3 (9.68%) |  |  |
| Ischemic Heart Disease | No | 97 (110.23%) | 73 (82.95%) | 24 (77.42%) | 1.42 (0.43-4.24) | 0.591 |
|  | Yes | 22 (25%) | 15 (17.05%) | 7 (22.58%) |  |  |
| Atrial Fibrillation | No | 98 (111.36%) | 72 (81.82%) | 26 (83.87%) | 0.87 (0.23-2.81) | 1 |
|  | Yes | 21 (23.86%) | 16 (18.18%) | 5 (16.13%) |  |  |
| Ever smoker | No | 32 (36.36%) | 26 (29.55%) | 6 (19.35%) | 1.74 (0.6-5.8) | 0.349 |
|  | Yes | 87 (98.86%) | 62 (70.45%) | 25 (80.65%) |  |  |
| Lesion side | Left | 36 (40.91%) | 27 (30.68%) | 9 (29.03%) | 1.37 (0.43-4.46) | 0.605 |
|  | Right | 35 (39.77%) | 24 (27.27%) | 11 (35.48%) |  |  |
| Age | 119 \| 88 \|31 | 67.8 (15.95) | 67.85 (15.4) | 67.4 (16.85) | -0.4 (-5.7-4.8) | 0.87 |
| NIHSS (stroke severity) | 119 \| 88 \| 31 | 2 (3) | 2 (3) | 1 (2.5) | 1 (0-1) | 0.065 |
| MADRS (depression) | 116 \| 86 \| 30 | 4 (7) | 4 (7) | 3.5 (7) | 0 (-1-2) | 0.72 |
| MoCA baseline | 119 \| 88 \| 31 | 25 (6) | 26 (5) | 25 (6) | 0 (-1-2) | 0.784 |
| MoCA 3 months | 119 \| 88 \| 31 | 27 (4) | 27 (4.25) | 26 (2) | 0 (-1-2) | 0.742 |
| MoCA 12 months | 119 \| 88 \| 31 | 26 (4) | 26 (4) | 26 (4) | 0 (-1-1) | 0.956 |
| Height | 108 \| 78 \| 30 | 170 (16) | 171.5 (15) | 166 (16.75) | 5 (0-10) | 0.048 |
| Weight | 111 \| 80 \| 31 | 78 (20) | 78.5 (20.75) | 76 (17.5) | 1 (-6-8) | 0.813 |
| BMI | 104 \| 78 \| 26 | 26.91 (4.73) | 26.9 (4.27) | 26.92 (6.23) | 0.13 (-1.86-2.04) | 0.913 |
| Systolic BP | 119 \| 88 \| 31 | 140 (25.5) | 136 (25.25) | 140 (35) | -3 (-13-6) | 0.577 |
| Diastolic BP | 119 \| 88 \| 31 | 77 (13) | 77 (14) | 76 (11.5) | 0 (-4-5) | 0.713 |
| RAPA (aerobic) | 119 \| 88 \| 31 | 4 (3) | 4 (3) | 4 (2) | 1 (0-2) | 0.065 |
| RAPA (strength) | 119 \| 88 \| 31 | 0 (1) | 0 (1) | 0 (0.5) | 0 (0-0) | 0.579 |
| Charlson Cmb. Index | 119 \| 88 \| 31 | 3 (2) | 3 (2) | 3 (2) | 0 (0-0) | 0.793 |
| *p-values from Fisher exact test or Wilcoxon rank-sum tests comparing those on medication or not  **BP**=Blood pressure; **BMI**=Body mass index; **Cmb**= Comorbidity; **MADRS**=Montgomery-Åsberg Depression Rating Scale; **MoCA**=Montreal Cognitive Assessment; **mRS**=modified Rankin Scale; **NIHSS**=National Institutes of Health Stroke Scale; **RAPA**=Rapid Assessment of Physical Activity; **TIA**=Transient Ischemic Attack; **tPA**= tissue-plasminogen activator | | | | | | |

##
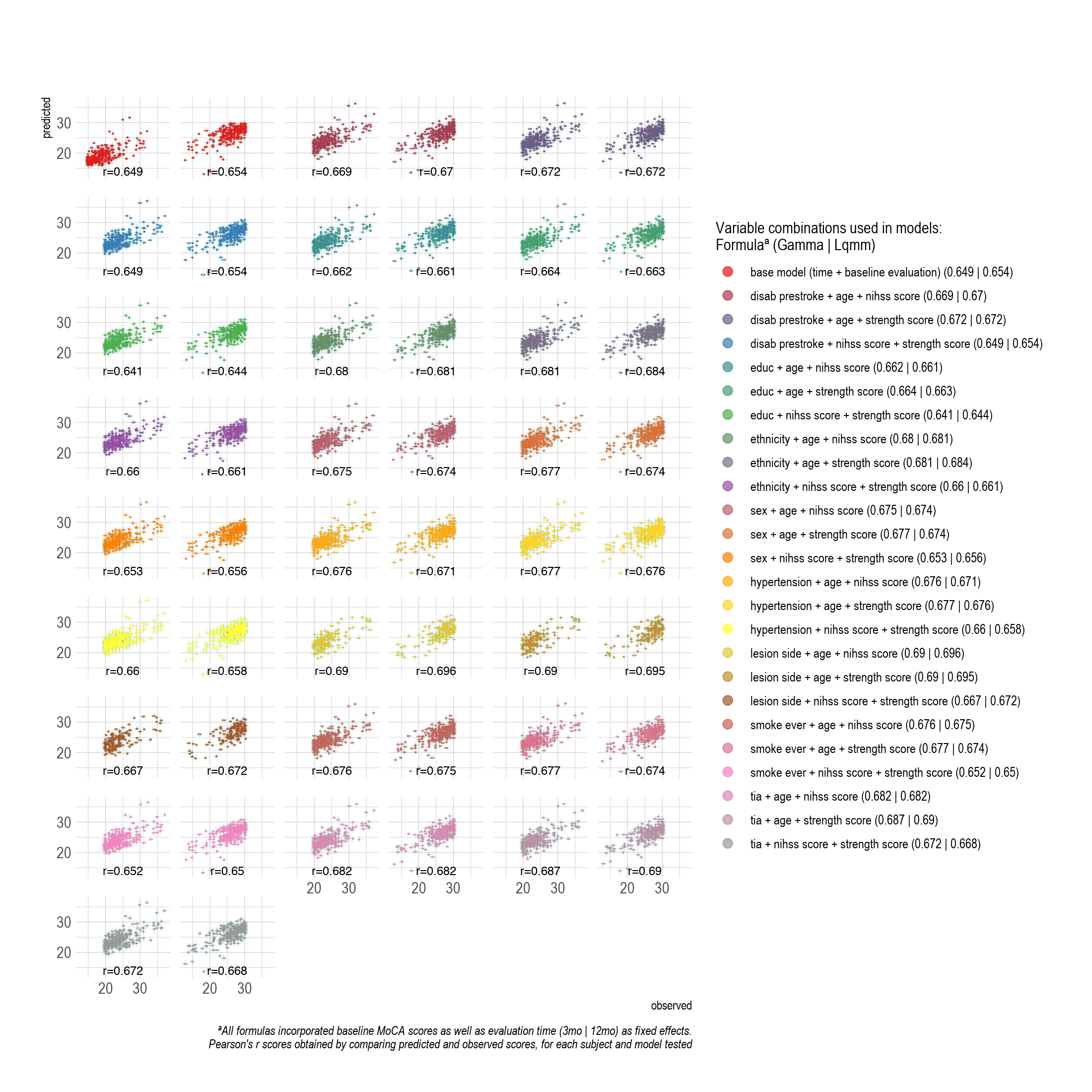
S1 Fig. Gamma versus linear quantile mixed method comparison for START cohort study

## S2 Fig. Gamma versus linear quantile mixed method comparison for Singapore cohort study


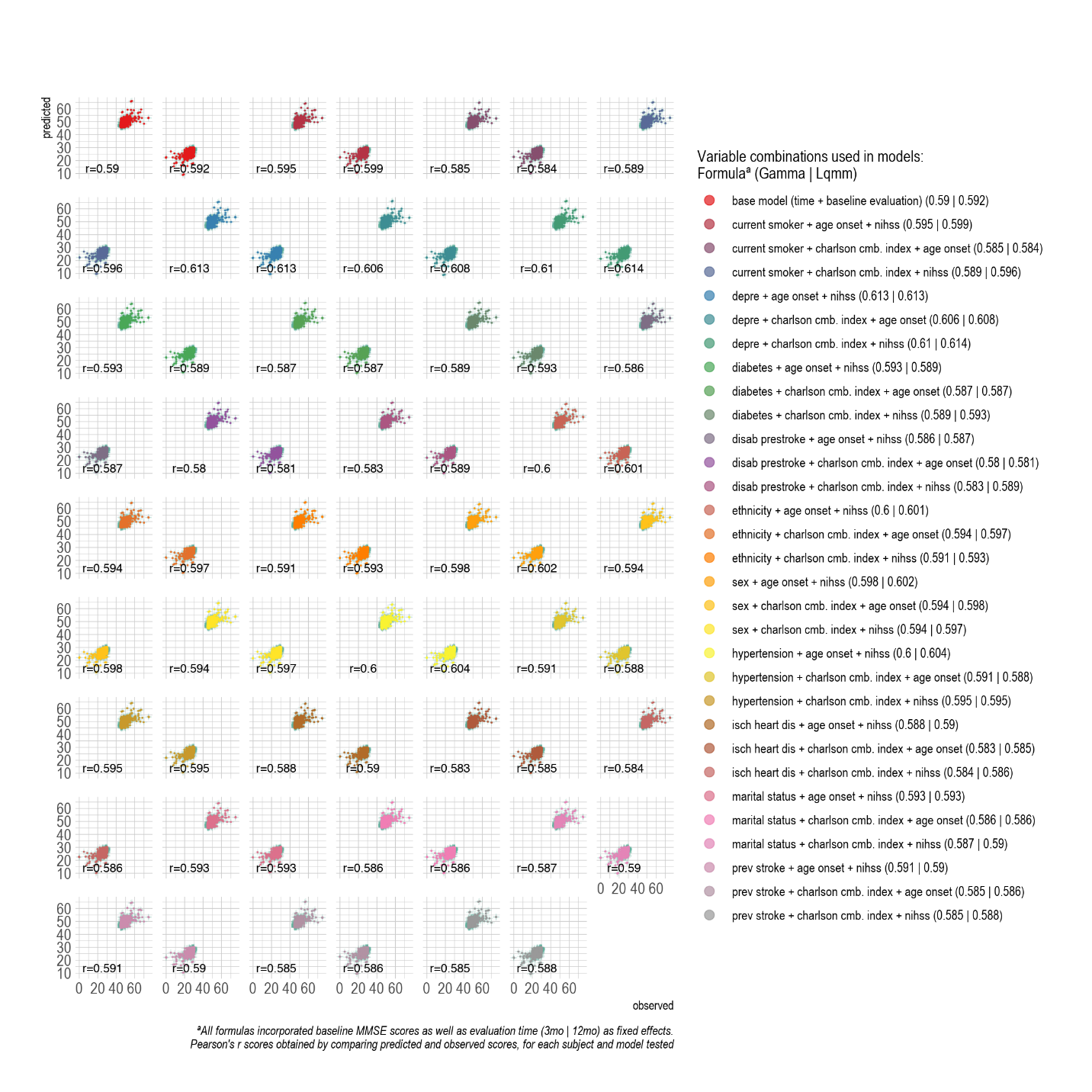


## S3 Fig. Improved-declined group (n=46) in START cohort. Individual trajectory of overall MoCA scores


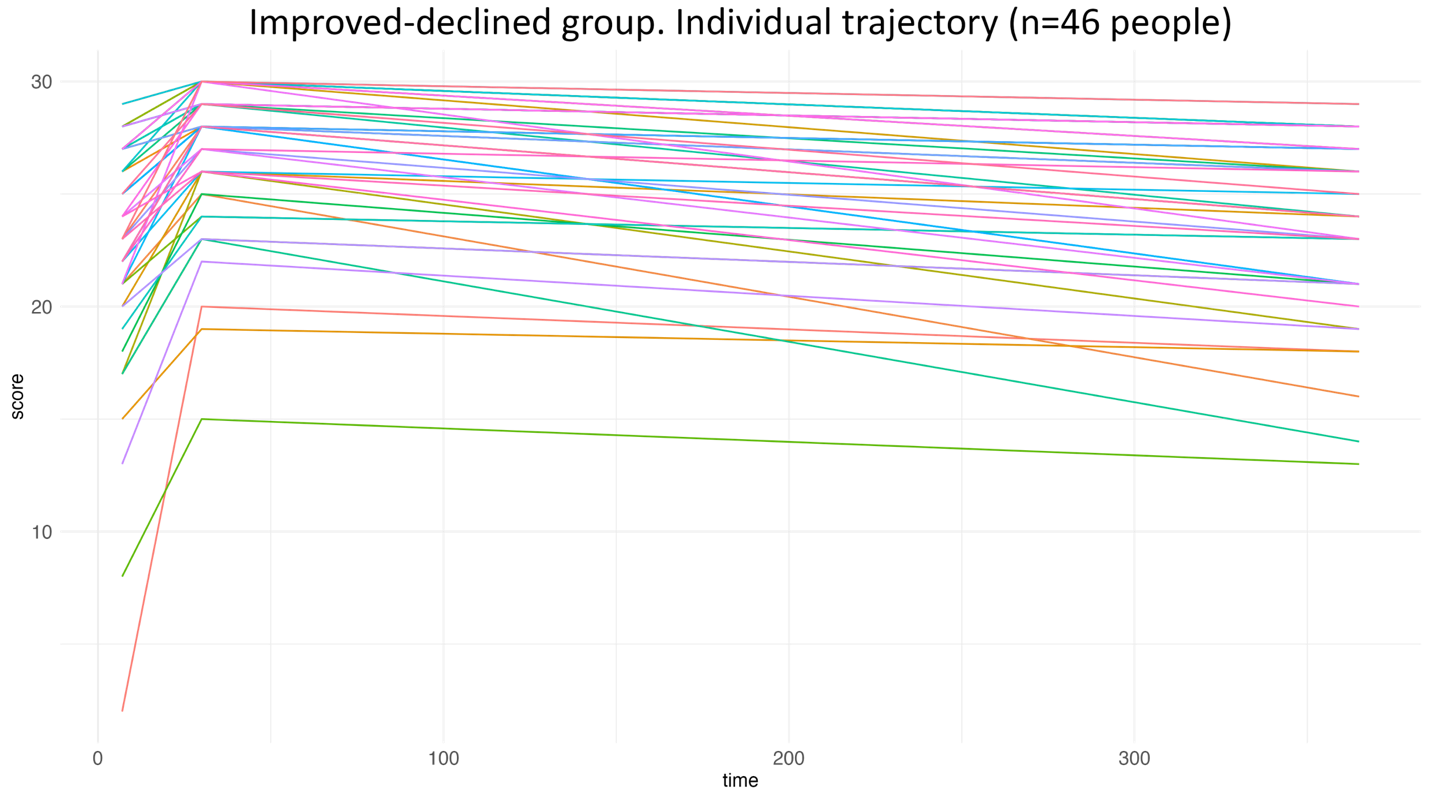

Supplement: S1 File — (DOCX) [file pone.0308103.s010.docx]
