# Supplementary material for: Characterization and individual-level prediction of cognitive state in the first year after ‘mild’ stroke
Source: PLoS One. 2024 Aug 30;19(8):e0308103. doi: 10.1371/journal.pone.0308103 (PMC11364298; doi:10.1371/journal.pone.0308103)
Supplement: Attachment — Submitted filename: Response-to-reviewers-R1-final.docx [file pone.0308103.s011.docx]

|  | **Reviewer’s comment** | **Authors’ response** | **Changes to the manuscript** |
| --- | --- | --- | --- |
| **Reviewer 1** |  |  |  |
| 1. | The publication does not provide any information regarding the infarct characteristics of the patients, including the infarct volume. The aforementioned characteristics can contribute to cognitive deterioration in certain individuals under observation. | We thank our reviewer for the thoughtful comments and observations.  We agree that multiple factors such as infarct volume, medications (including tPA), hemorrhagic transformation, and blood indicators of vitamin B12 and cholesterol levels can contribute to cognitive status. Here, our focus is on the characterization of the cognitive trajectory after mild stroke. Specifically, we chose to investigate demographic (e.g. age), clinically accessible data (e.g. stroke severity), and modifiable behavioral factors (e.g. physical activity levels), given their role as robust predictors of cognition and recovery, and their potential to have value as individual predictors.  While we did measure some of the factors mentioned by the reviewer, and have included these as additional background information where available, it was not our purpose nor do we have the power with our sample size to systematically investigate the contributions of these factors. For example, infarct volume was only recorded for the subjects who underwent the EXTEND Randomized Controlled Trial (RCT; 12 individuals in our dataset).  In response to this comment, we have therefore mentioned the factors above in our introduction and further clarified the purpose of our modelling approach as shown in column 3.  We also conducted one additional analysis related to tPA, as indicated in column 3, and discussed below in response to comment 2. | **Please see changes to manuscript as follows:**  **Abstract line 30**: “… combination of demographic, clinical, and lifestyle factors predicting individual-level cognitive state at 3- and 12-months after mild stroke …”  **Introduction (lines 75-85)**  “A range of factors have potential to influence cognition and its trajectory post-stroke. These include: demographic, such as age, education and gender [15]; clinical, including initial severity of cognitive impairment, stroke severity [16]; neurological, including lesion side and volume [17]; interventions such as tissue-plasminogen activator (i.e. tPA or alteplase), anticoagulant or antiplatelet medications and their association with hemorrhagic transformation [18]; modifiable risk factors such as diet and lifestyle [19]; and blood-based biomarkers (e.g. cholesterol, vitamin B12, vitamin D, and inflammatory biomarkers) [10,20,21]. In the present study we focus on three main predictor categories: demographic characteristics, clinical characteristics, and lifestyle factors, given the consistent evidence found for these variables, the available data, and the potential to identify modifiable factors in this cohort of mild stroke survivors”  **Methods (lines 165-184). We have created a new figure to visually explain our modelling approach, and reworded our statistical analysis section for further clarity:**  “A visual guide explaining the variable selection process we completed as part of our exploratory analyses is depicted in Figure 1. The selection of potential variables for our final model validation involved an exploratory phase in which we employed unadjusted and adjusted analyses, using groups of variables from three predictor categories (i.e. demographic characteristics, clinical characteristics, and lifestyle factors). The skewed nature of the primary outcome (i.e. overall MoCA score), led us in the first instance to select binary and quantile regressions, in line with previous literature [11,12]. For these models, each baseline variable from Table 1 was studied, one at a time, to explore their predictive association with cognition at each time-point (i.e. baseline, 3 and 12-months).  Two additional model formulations were used in this exploratory phase to account for the distribution of our outcome data and the repeated measurements: linear-quantile mixed-models (LQMM) [43] and Gamma-distributed mixed-linear regression models (henceforth called “mixed Gamma models”) [44,45]. These two modeling approaches take into account both the skewed nature of the overall MoCA scores, as well as the follow-up (i.e. longitudinal) measurements recorded for each participant. For each model formulation, we conducted additional exploratory, unadjusted, bivariate analyses, using overall MoCA scores (all 3 time-points at once) as the outcome (i.e. dependent) variable; and one predictor (i.e. independent) variable from Table 1, introduced one at a time. The same models were then re-run adjusting for baseline MoCA score to predict subsequent MoCA scores at 3 and 12-months post-stroke.”  **We have also added further clarification in the subsection below (lines 185–200)**  **“Identifying the ‘best’ combinations of predictive variables for cognition, based on individual-patient prediction**  After finalizing our exploratory analyses, a set of candidate multivariate formulae were constructed, combining the baseline variables that showed a significant association with MoCA in the unadjusted (i.e. bivariate), and adjusted analyses (Table S1). These multivariate formulae were limited to a maximum of three predictors (i.e. one categorical, plus two continuous), adjusting for baseline MoCA scores each time. Pseudo-replication was controlled for by including each participant as a random intercept (i.e. random effect) in all models. Each model was fitted using the ‘k-fold’ cross-validation, also known as ‘model training’ method [46], which consists of fitting a model iteratively after removing one participant in each iteration. The resulting model is then tested on the held-out individual to predict their scores. This way, each individual has the opportunity to be the ‘test’ data.  After predicting scores for each individual person with all combinations of candidate baseline variables, we compared predicted and observed scores for each individual. Overall best predictive performance was arrived at by calculating a Pearson’s correlation between predicted and observed scores for each individual in each model. |
| 2. | The text does not provide any mention of the treatment strategies. The inquiry that arose in my thoughts was regarding the number of patients who received tPA, anticoagulant, or antiplatelet medication. Furthermore, it is well-established that the administration of statin medicine can lead to cognitive impairments. In order to mitigate the potential impact of drugs, it is imperative to take the these factors into consideration. | Thank you. We have now included details on the number of stroke survivors who received tPA, anticoagulant, or antiplatelet medication in the text and in Supplementary Table S6  To address the reviewer’s comment, we compared those participants on tPA, anticoagulant or antiplatelet medication for any reason (n=31 participants, which includes those who were in the EXTEND RCT and received tPA treatment i.e. n=6), versus those who did not receive any of those medications (n=88 participants). Our analyses yielded no significant differences in overall cognition between the two groups of participants at baseline, 3 or 12 months; and no differences in baseline variables. Please refer to Table S6 for more details | **We have included the following statement in our Risk of Bias section (lines 146-148):**  “In addition, we conducted comparative post-hoc analyses to study potential effect modifiers from the use of medication such as antiplatelet, tPA, or anticoagulant during the course of the study ”  **In the results section (lines 308–315), we have reported on this analysis:**  “**Impact of tPA, anticoagulant or antiplatelet medication on cognition.**  Lastly, we compared those participants on tPA, anticoagulant or antiplatelet medication for any reason (n=31 participants, which includes those who were in the EXTEND RCT and received tPA treatment i.e. n=6), versus those who did not receive any of those medications (n=88 participants) throughout the study. Our post-hoc analyses yielded no significant differences in overall cognition between the two groups of participants at baseline, 3- or 12-months; and no significant differences in baseline variables. Please refer to Table S6 for more details.”  **Please also refer to new analysis in Supplementary Table S6 titled:**  Table S6 – Comparison of START participants on antiplatelet, tPA, or anticoagulant medication (n=31) versus those not on those medications (n=88) |
| 3. | It is also imperative to ascertain whether the patients had **hemorrhagic transformation**, as this phenomenon has the potential to induce a fast increase in the National Institutes of Health Stroke Scale (NIHSS) score and important to acknowledge the preceding instances of COVID-19 infections since COVID-19 is a subject of extensive investigation and is considered a potential cause of cognitive decline. | Unfortunately, we do not have comprehensive data on hemorrhagic transformation readily available for this cohort, nor a sample size able to include this in our predictive model. We do however acknowledge the importance of this phenomenon and potential impact of vascular vulnerabilities and hemorrhagic transformation on cognition and cognitive decline after stroke (Rost et al., 2021).  We have therefore included this as a limitation and highlighted the importance of haemorrhagic transformation in the discussion.  In relation to COVID, although our cohort data was collected prior to COVID, the reviewer may be interested to learn that a few of our team members contributed blood samples from our cohort, given vascular vulnerability of stroke survivors, and indeed saw changes in fibrin clot characteristics and anticoagulant response in our SARS-CoV-2 infected endothelial model (McCfferty et al., 2022).  McCafferty, C., Lee, L., Cai1, T., Praporski, S., Stolper, J., Karlaftis, V., Attard, C., Myint, D., **Carey, L.M**., Howells, D.W., **Donnan, G.A**., **Davis, S., Ma, H.**, Crewther, S., Nguyen, V.A., Van Den Helm, S., Letunica, N., Swaney, E., Elliott, D., Subbarao, K., Ignjatovic, V., Monagle, P. (2022). Fibrin clot characteristics and anticoagulant response in a SARS-CoV-2 infected endothelial model. *eJHaem.* DOI: 10.1002/jha2.407. Article ID: JHA2407 DOI: 10.1002/jha2.407  Rost, N. S., Meschia, J. F., Gottesman, R., Wruck, L., Helmer, K., Greenberg, S. M., & DISCOVERY Investigators (2021). Cognitive Impairment and Dementia After Stroke: Design and Rationale for the DISCOVERY Study. *Stroke*, *52*(8), e499–e516. https://doi.org/10.1161/STROKEAHA.120.031611 | **We have included this important observation in our limitations (lines 424 to 427), which now read:**  “Although our study did not include a comprehensive examination of vascular vulnerabilities and hemorrhagic transformation on cognition, we acknowledge the importance of monitoring these variables and including them in future studies that set out to understand the many facets of post-stroke cognitive trajectory [63,64]” |
| 4. | It is also important to include and adjust patients' **blood work, such as vitamin B12, cholesterol levels, and vitamin D** which are crucial parameters to lead to cognitive detoriation. | Thank you. We have previously reported on blood based biomarkers of recovery post-stroke in this cohort (Nguyen et al., 2020a; Nguyen et al., 2020b) with white blood cell (WBC) and neurotrophil count (NC) taken between 24 and 48 hours emerging as being predictive of cognitive outcome post-stroke.  We now refer to this work in the introduction.  Nguyen VA, Crewther SG, Howells DW, Wijeratne TK, Ma H, Hankey GJ, **Davis S, Donnan GA**, **Carey LM**, and the START research team. (2020a). Acute routine leukocyte and neutrophil counts are predictive of post-stroke recovery at 3- and 12-months post-stroke: An exploratory study. *Neurorehabilitation and Neural Repair*. 34:844-855. [doi.org/10.1177/1545968320948607](https://doi.org/10.1177%2F1545968320948607).  Nguyen VA, Riddell N, Crewther SG, Faou P, Rajapaksha H, Howells DW, Hankey GJ, Wijeratne TK, **Ma H**, **Davis S, Donnan GA**, **Carey LM**. (2020b) Longitudinal Ischemic Stroke Recovery Dominated by Dysregulation of Complement System – A Proteomics Pathway Analysis. *Frontiers in Neurology* [doi.org/10.3389/fneur.2020.00692](https://doi.org/10.3389/fneur.2020.00692). | **See changes in the Introduction of the manuscript (lines 75–85, especially 80-82)**  ...and blood-based biomarkers (i.e. cholesterol, vitamin B12, vitamin D, and concentration of plasma inflammatory biomarkers) [10,20,21]. |
| **Reviewer 2** |  |  |  |
|  | No revisions requested |  |  |
| **Reviewer 3** |  |  |  |
| 1. | Line 131: Grammatical error: Fisher’s exact tests. | Thanks for noting this grammatical error. We have fixed it in the revised version of our manuscript | **Please refer to manuscript line 149–151 which now read:**  “Baseline characteristics between included and excluded participants were summarized and compared using Wilcoxon rank-sum or Fisher’s exact tests, to compare continuous and categorical variables, respectively.” |
| 2. | Line 171: State the alpha level which attained 80% power. | We have now indicated the alpha level in our manuscript | **Please refer to lines 202–205:**  “Given our smaller sample size, in our pre-analysis phase, we simulated a GLMM model, using a Gamma distribution for a maximum of five predictors, with 0.8 power, and alpha (p) set at 0.05” |
| 3. | Explain all abbreviations at first mention. | Thank you. We have carefully reviewed the manuscript and now include all abbreviations at first mention. | **We have checked our work and corrected the following:**  Line 94: The STroke imAging pRevention and treatment (START)  Lines 95-96: START-Extending the time for Thrombolysis in Emergency Neurological Deficits (EXTEND)  Lines 97–98: START- Prediction and Prevention to Achieve Optimal Recovery Endpoints after stroke (PrePARE) cohort study  Line 124–125: Montgomery-Åsberg Depression Rating Scale, MADRS  Line 125–126: National Institutes of Health Stroke Scale, NIHSS  Line 442: Commonwealth Scientific and Industrial Research Organisation (CSIRO)  Line 443–444: National Health and Medical Research Council (NHMRC)  Line 444–445: La Trobe University (La Trobe Full-Fee research, LTUFFR, and La Trobe Postgraduate Research, LTUPR scholarships). |
| 4. | Indicate if p-values were adjusted for multiple comparisons, specifically those in Table 2. | We thank our reviewer for this observation. The p-values displayed in Table 2 come from a comparison between two medians and been adjusted for multiple comparison using the Benjamini and Hochber method. | Please see changes to manuscript in Table 2  P values have been updated (highlighted) and the following text included as a footnote to the table:  … All p-values were adjusted for multiple comparisons using the Benjamini and Hochberg [66] method. |
